# Supplementary material for: Comparison of Different d-SPE Sorbent Performances Based on Quick, Easy, Cheap, Effective, Rugged, and Safe (QuEChERS) Methodology for Multiresidue Pesticide Analyses in Rapeseeds
Source: Molecules. 2021 Nov 6;26(21):6727. doi: 10.3390/molecules26216727 (PMC8588138; doi:10.3390/molecules26216727)
Supplement: Supplementary file 1 [file molecules-26-06727-s001.zip › molecules-1453470-supplementary.pdf]

## Tables

Table S1: Names, CAS number, Log P and HPLC-MS/MS acquisition parameters of studied pesticides and contaminants

| Pesticides | $R_t$ (min) | CAS number | Log P | Quantification transition | | | | | Confirmation transition | | | | |
|---|---|---|---|---|---|---|---|---|---|---|---|---|---|
| | | | | Precursor ions (*m/z*) | Fragment ions (*m/z*) | DP (V) | CE (V) | CXP (V) | Precursor ions (*m/z*) | Fragment ions (*m/z*) | DP (V) | CE (V) | CXP (V) |
| Acephate | 3.91 | 30650-19-1 | -0.85 | 184 | 143 | 66 | 13 | 12 | 184 | 125 | 66 | 25 | 22 |
| Acetochlor | 5.53 | 34256-82-1 | 3.03 | 270 | 224 | 56 | 11 | 12 | 270 | 148 | 56 | 29 | 8 |
| Acibenzolar-S- methyl | 6.41 | 135158-54-2 | 3.01 | 211 | 136 | 46 | 39 | 6 | 211 | 140 | 46 | 31 | 6 |
| Alachlor | 6.72 | 15972-60-8 | 3.52 | 270 | 162 | 71 | 25 | 8 | 270 | 45 | 71 | 27 | 10 |
| Aldicarb sulfone | 3.02 | 1646-88-4 | -0,57 | 223 | 86 | 76 | 29 | 14 | 223 | 148 | 76 | 13 | 14 |
| Ametoctradin | 7.85 | 865318-97-4 | 4. 40 | 276 | 70 | 26 | 81 | 10 | 276 | 43 | 26 | 103 | 8 |
| Ametryn | 5.43 | 834-12-8 | 2.98 | 228 | 186 | 60 | 37 | 14 | 228 | 96 | 60 | 35 | 16 |
| Anilofos | 6.92 | 64249-01-0 | 3.81 | 368 | 199 | 66 | 19 | 18 | 368 | 125 | 66 | 45 | 18 |
| Azaconazole | 4.37 | 60207-31-0 | 2.32 | 302 | 161 | 56 | 39 | 10 | 302 | 231 | 56 | 23 | 12 |
| Azinphos-methyl | 5.02 | 86-50-0 | 2.75 | 318 | 261 | 71 | 11 | 20 | 318 | 132 | 71 | 21 | 8 |
| Aziprotryne | 5.92 | 4658-28-0 | 3.00 | 226 | 156 | 76 | 21 | 12 | 226 | 125 | 76 | 17 | 14 |
| Azoxystrobin | 5.55 | 131860-33-8 | 2.50 | 404 | 372 | 66 | 21 | 10 | 404 | 344 | 66 | 29 | 18 |
| Barban | 7.24 | 101-27-9 | 3.41 | 258 | 143 | 86 | 29 | 10 | 258 | 178 | 86 | 13 | 12 |
| Benalaxyl | 6.79 | 71626-11-4 | 3.40 | 326 | 148 | 76 | 27 | 8 | 326 | 208 | 76 | 21 | 10 |
| Benoxacor | 7.34 | 98730-04-2 | 2.70 | 262 | 149 | 156 | 25 | 16 | 262 | 134 | 156 | 39 | 14 |
| Bifenox | 9.21 | 42576-02-3 | 4.48 | 359 | 310 | 64 | 17 | 18 | 359 | 189 | 64 | 35 | 20 |
| Bixafen | 6.46 | 581809-46-3 | 4.70 | 415 | 395 | 126 | 21 | 24 | 415 | 267 | 126 | 33 | 18 |
| Boscalid | 7.15 | 188425-85-6 | 2.96 | 343 | 307 | 106 | 29 | 18 | 343 | 140 | 106 | 29 | 12 |
| Bupirimate | 7.64 | 41483-43-6 | 2.70 | 317 | 166 | 71 | 33 | 16 | 317 | 237 | 71 | 27 | 18 |
| Buprofezin | 9.04 | 69327-76-0 | 4.30 | 306 | 201 | 56 | 17 | 14 | 306 | 116 | 56 | 23 | 20 |
| Chlorfenvinphos | 8.40 | 470-90-6 | 3.81 | 359 | 155 | 69 | 19 | 8 | 359 | 99 | 69 | 43 | 16 |
| Chlorobromuron | 8.51 | 13360-45-7 | 3.09 | 295 | 206 | 136 | 27 | 20 | 293 | 203 | 136 | 31 | 12 |
| Chlorfluazuron | 10.32 | 71422-67-8 | 5.80 | 542 | 385 | 176 | 33 | 18 | 542 | 158 | 176 | 27 | 8 |
| Chlorpyrifos | 9.11 | 2921-88-2 | 4.96 | 350 | 97 | 86 | 41 | 6 | 350 | 198 | 86 | 25 | 10 |

| | | | | | | | | | | | | | |
|---|---|---|---|---|---|---|---|---|---|---|---|---|---|
| Chlorpyrifos-methyl | 9.23 | 5598-13-0 | 4.31 | 322 | 125 | 71 | 27 | 6 | 322 | 290 | 71 | 25 | 16 |
| Cinosulfuron | 4.76 | 94593-91-6 | 2.04 | 414 | 183 | 81 | 23 | 10 | 414 | 215 | 81 | 21 | 10 |
| Climbazole | 5.94 | 38083-17-9 | 3.70 | 293 | 197 | 111 | 23 | 24 | 293 | 69 | 111 | 59 | 16 |
| Clodinafop-propargyl | 6.02 | 105512-06-9 | 3.90 | 350 | 266 | 104 | 21 | 14 | 350 | 91 | 104 | 41 | 18 |
| Clofentezine | 5.87 | 74115-24-5 | 3.10 | 303 | 138 | 60 | 27 | 10 | 303 | 102 | 60 | 59 | 18 |
| Cloquintocet-mexyl | 7.32 | 99607-70-2 | 5.03 | 336 | 238 | 91 | 27 | 12 | 336 | 192 | 91 | 41 | 12 |
| Coumaphos | 6.51 | 56-72-4 | 4.13 | 363 | 227 | 101 | 35 | 12 | 363 | 307 | 101 | 23 | 16 |
| Coumatetralyl | 5.46 | 5836-29-3 | 3.46 | 293 | 175 | 66 | 45 | 16 | 293 | 141 | 66 | 41 | 12 |
| Crufomate | 8.76 | 299-86-5 | 3.42 | 292 | 236 | 96 | 27 | 14 | 292 | 108 | 96 | 39 | 6 |
| Cyanazine | 7.11 | 21725-46-2 | 2.22 | 241 | 214 | 81 | 25 | 12 | 241 | 243 | 81 | 25 | 14 |
| Cycloxydim | 6.21 | 101205-02-1 | 3.20 | 326 | 280 | 101 | 19 | 16 | 326 | 180 | 101 | 29 | 12 |
| Cyflumetofen | 5.98 | 400882-07-7 | 4.30 | 465 | 173 | 121 | 33 | 10 | 465 | 249 | 121 | 53 | 32 |
| Cymiazole | 2.96 | 61676-87-7 | 0.60 | 219 | 171 | 86 | 37 | 14 | 219 | 144 | 86 | 43 | 8 |
| Cyproconazole | 4.67 | 94361-06-5 | 2.90 | 292 | 70 | 81 | 35 | 10 | 292 | 125 | 81 | 35 | 6 |
| Cyprodinil | 9.58 | 121552-61-2 | 4.00 | 226 | 93 | 84 | 47 | 16 | 226 | 91 | 84 | 49 | 16 |
| Demeton-S-methyl sulfone | 3.48 | 17040-19-6 | -0.30 | 263 | 169 | 61 | 23 | 14 | 263 | 121 | 61 | 23 | 10 |
| Desmetryn | 5.86 | 1014-69-3 | 2.38 | 214 | 172 | 126 | 23 | 10 | 214 | 124 | 126 | 59 | 20 |
| Diafenthiuron | 10.05 | 80060-09-9 | 6.00 | 385 | 329 | 26 | 25 | 20 | 385 | 270 | 26 | 19 | 18 |
| Dialifos | 7.76 | 10311-84-9 | 4.69 | 394 | 208 | 39 | 23 | 10 | 394 | 187 | 39 | 13 | 12 |
| Diazinon | 7.43 | 333-41-5 | 3.81 | 305 | 169 | 71 | 29 | 8 | 305 | 97 | 71 | 41 | 6 |
| Diclofop-methyl | 6.92 | 51338-27-3 | 4.62 | 358 | 281 | 64 | 21 | 16 | 358 | 120 | 64 | 39 | 8 |
| Diethofencarb | 5.67 | 87130-20-9 | 2.91 | 268 | 226 | 81 | 15 | 12 | 268 | 180 | 81 | 23 | 10 |
| Difenoconazole | 8.54 | 119446-68-3 | 4.30 | 406 | 251 | 66 | 37 | 14 | 406 | 337 | 66 | 23 | 18 |
| Diflufenican | 7.78 | 83164-33-4 | 4.90 | 395 | 266 | 66 | 33 | 16 | 395 | 246 | 66 | 49 | 16 |
| Dimethachlor | 9.08 | 50563-36-5 | 2.17 | 256 | 224 | 86 | 21 | 14 | 256 | 148 | 86 | 33 | 8 |
| Dimethenamid | 7.63 | 87674-68-8 | 2.15 | 276 | 244 | 44 | 29 | 10 | 276 | 168 | 44 | 33 | 10 |
| Dimethomorph | 6.70 | 110488-70-5 | 2.68 | 388 | 301 | 91 | 29 | 18 | 388 | 165 | 91 | 43 | 8 |
| Dimoxystrobin | 5.56 | 149961-52-4 | 3.90 | 327 | 205 | 56 | 15 | 12 | 327 | 116 | 56 | 57 | 14 |
| Dioxathion | 4.89 | 78-34-2 | 3.00 | 474 | 271 | 96 | 19 | 14 | 474 | 97 | 96 | 61 | 6 |
| Dipropetryn | 6.87 | 4147-51-7 | 3.81 | 256 | 214 | 51 | 31 | 12 | 256 | 144 | 51 | 37 | 10 |
| Ditalimphos | 7.67 | 5131-24-8 | 3.48 | 300 | 148 | 76 | 29 | 26 | 300 | 130 | 76 | 47 | 10 |
| Emamectin Ba | 10.78 | 155569-91-8 | 5.00 | 886 | 158 | 36 | 43 | 10 | 886 | 82 | 36 | 127 | 12 |

| | | | | | | | | | | | | | |
|---|---|---|---|---|---|---|---|---|---|---|---|---|---|
| Emamectin Bb | 10.03 | 155569-91-8 | 3.70 | 886 | 158 | 36 | 43 | 10 | 886 | 82 | 36 | 127 | 12 |
| Epoxiconazole | 8.91 | 133855-98-8 | 3.44 | 330 | 121 | 81 | 27 | 6 | 330 | 101 | 81 | 63 | 6 |
| Etaconazole | 9.12 | 60207-93-4 | 3.10 | 329 | 159 | 86 | 37 | 12 | 329 | 55 | 86 | 47 | 8 |
| Ethion | 10.73 | 563-12-2 | 5.07 | 385 | 199 | 36 | 17 | 12 | 385 | 171 | 36 | 23 | 8 |
| Ethofumesate | 4.00 | 26225-79-6 | 2.70 | 287 | 121 | 81 | 23 | 8 | 287 | 161 | 81 | 27 | 10 |
| Ethoprophos | 7.34 | 13194-48-4 | 3.59 | 243 | 131 | 56 | 29 | 6 | 243 | 97 | 56 | 41 | 10 |
| Etoxazole | 6.75 | 153233-91-1 | 5.59 | 360 | 141 | 71 | 41 | 14 | 360 | 113 | 71 | 79 | 8 |
| Etrimfos | 4.51 | 38260-54-7 | 3.20 | 293 | 125 | 81 | 33 | 6 | 293 | 265 | 81 | 21 | 14 |
| Famoxadone | 3.79 | 131807-57-3 | 4.65 | 392 | 331 | 61 | 15 | 18 | 392 | 238 | 61 | 23 | 12 |
| Fenamiphos | 6.00 | 22224-92-6 | 3.23 | 304 | 217 | 81 | 31 | 12 | 304 | 202 | 81 | 45 | 10 |
| Fenarimol | 7.65 | 60168-88-9 | 3.65 | 331 | 268 | 86 | 33 | 18 | 331 | 81 | 86 | 47 | 4 |
| Fenazaquin | 9.76 | 120928-09-8 | 5.51 | 307 | 161 | 56 | 31 | 8 | 307 | 147 | 56 | 25 | 8 |
| Fenbuconazole | 8.09 | 114369-43-6 | 3.23 | 337 | 125 | 76 | 37 | 6 | 337 | 70 | 76 | 33 | 6 |
| Fenpropidin | 7.95 | 67306-00-7 | 5.50 | 274 | 147 | 76 | 37 | 8 | 374 | 117 | 76 | 65 | 6 |
| Fenpropimorph | 9.12 | 67564-91-4 | 4.93 | 304 | 147 | 91 | 39 | 8 | 304 | 117 | 91 | 71 | 8 |
| Fenpyroximate | 5.69 | 134098-61-6 | 5. 01 | 422 | 366 | 126 | 23 | 10 | 422 | 135 | 126 | 41 | 8 |
| Fensulfothion | 4.09 | 115-90-2 | 2.23 | 309 | 281 | 66 | 17 | 16 | 309 | 253 | 66 | 25 | 16 |
| Fensulfothion-oxon | 5.32 | 6552-21-2 | 1.40 | 264 | 231 | 101 | 23 | 14 | 264 | 216 | 101 | 33 | 14 |
| Fensulfothion-sulfone | 4.63 | 14255-72-2 | 2.56 | 325 | 269 | 51 | 21 | 16 | 325 | 297 | 51 | 17 | 18 |
| Fenthion | 2.78 | 55-38-9 | 4.09 | 279 | 247 | 66 | 19 | 16 | 279 | 169 | 66 | 17 | 14 |
| Fenthion sulfone | 4.42 | 3761-42-0 | 2.80 | 311 | 125 | 101 | 29 | 8 | 311 | 279 | 101 | 27 | 16 |
| Fenthion sulfoxide | 3.25 | 3761-41-9 | 2.70 | 295 | 280 | 96 | 25 | 16 | 295 | 127 | 96 | 41 | 22 |
| Flamprop-isopropyl | 7.39 | 52756-22-6 | 3.69 | 364 | 105 | 64 | 15 | 12 | 364 | 304 | 64 | 17 | 8 |
| Flazasulfuron | 3.15 | 104040-78-0 | 1.08 | 408 | 182 | 96 | 23 | 12 | 408 | 227 | 96 | 25 | 10 |
| Florasulam | 3.12 | 145701-23-1 | 1.22 | 360 | 129 | 80 | 29 | 8 | 360 | 192 | 80 | 23 | 10 |
| Flubendiamide | 6.74 | 272451-65-7 | 4.20 | 683 | 408 | 176 | 13 | 20 | 683 | 256 | 176 | 85 | 14 |
| Flucycloxuron | 7.46 | 94050-52-9 | 6.10 | 484 | 132 | 71 | 45 | 22 | 484 | 169 | 71 | 57 | 22 |
| Flufenacet | 5.85 | 142459-58-3 | 3.20 | 364 | 194 | 44 | 17 | 12 | 364 | 152 | 44 | 27 | 8 |
| Flufenoxuron | 9.54 | 101463-69-8 | 6.16 | 489 | 158 | 71 | 27 | 8 | 489 | 141 | 71 | 57 | 6 |
| Fluopicolide | 5.45 | 239110-15-7 | 3.26 | 385 | 175 | 71 | 33 | 14 | 385 | 173 | 71 | 33 | 14 |
| Flurochloridon | 4.87 | 61213-25-0 | 3.36 | 312 | 292 | 76 | 29 | 18 | 312 | 145 | 76 | 67 | 8 |
| Flurprimidol | 4.42 | 56425-91-3 | 3.34 | 313 | 270 | 36 | 33 | 16 | 313 | 201 | 36 | 23 | 12 |
| Flurtamone | 4.59 | 96525-23-4 | 4.50 | 334 | 247 | 81 | 39 | 14 | 334 | 178 | 81 | 67 | 10 |

| | | | | | | | | | | | | | |
|---|---|---|---|---|---|---|---|---|---|---|---|---|---|
| Flusilazole | 6.29 | 85509-19-9 | 3.70 | 316 | 247 | 106 | 27 | 14 | 316 | 169 | 106 | 25 | 14 |
| Fluxapyroxad | 7.21 | 907204-31-3 | 3.70 | 382 | 362 | 166 | 19 | 16 | 382 | 342 | 166 | 29 | 32 |
| Fluxastrobin | 5.62 | 887973-21-9 | 2.86 | 460 | 428 | 81 | 25 | 12 | 460 | 189 | 81 | 19 | 26 |
| Fonofos | 6.08 | 944-22-9 | 3.94 | 247 | 137 | 96 | 15 | 8 | 247 | 109 | 96 | 25 | 6 |
| Foramsulfuron | 2.00 | 173159-57-4 | -0.78 | 453 | 182 | 61 | 27 | 12 | 453 | 272 | 61 | 21 | 16 |
| Forchlofenuron | 4.56 | 68157-60-8 | 3.20 | 248 | 129 | 111 | 23 | 12 | 248 | 155 | 111 | 23 | 10 |
| Furmecyclox | 8.46 | 60568-05-0 | 3.10 | 252 | 170 | 56 | 17 | 12 | 252 | 129 | 56 | 31 | 16 |
| Haloxyfop-methyl | 8.73 | 69806-40-2 | 4.07 | 376 | 316 | 129 | 23 | 18 | 376 | 288 | 129 | 33 | 14 |
| Heptenophos | 5.41 | 23560-59-0 | 2.32 | 251 | 127 | 96 | 23 | 22 | 251 | 109 | 96 | 37 | 6 |
| Hexazinone | 4.32 | 51235-04-2 | 1.85 | 253 | 171 | 71 | 21 | 8 | 253 | 71 | 71 | 47 | 4 |
| Hexythiazox | 8.56 | 78587-05-0 | 5.57 | 353 | 228 | 71 | 23 | 14 | 353 | 168 | 71 | 33 | 12 |
| Imazalil | 6.39 | 35554-44-0 | 3.82 | 297 | 159 | 66 | 31 | 14 | 297 | 201 | 66 | 23 | 10 |
| Imazosulfuron | 3.77 | 122548-33-8 | 2.43 | 413 | 153 | 61 | 19 | 10 | 413 | 156 | 61 | 29 | 10 |
| Imibenconazole | 5.22 | 86598-92-7 | 4.94 | 411 | 125 | 86 | 43 | 8 | 411 | 171 | 86 | 27 | 10 |
| Indoxacarb | 6.00 | 144171-61-9 | 4.65 | 528 | 293 | 64 | 21 | 18 | 528 | 203 | 64 | 51 | 12 |
| Ipconazole | 7.43 | 125225-28-7 | 4.21 | 334 | 70 | 151 | 67 | 10 | 334 | 125 | 151 | 61 | 16 |
| Isofenphos | 8.81 | 25311-71-1 | 4.12 | 346 | 245 | 86 | 17 | 14 | 346 | 217 | 86 | 33 | 16 |
| Isofenphos-methyl | 8.02 | 99675-03-3 | 3.80 | 332 | 231 | 61 | 19 | 20 | 332 | 273 | 61 | 7 | 14 |
| Isopyrazam | 5.69 | 881685-58-1 | 4.00 | 360 | 244 | 111 | 35 | 18 | 360 | 320 | 111 | 31 | 16 |
| Isoxaflutole | 4.09 | 141112-29-0 | 2.32 | 360 | 251 | 31 | 21 | 8 | 360 | 220 | 31 | 27 | 18 |
| Kresoxim-methyl | 5.32 | 143390-89-0 | 3.40 | 331 | 116 | 66 | 25 | 20 | 314 | 206 | 66 | 13 | 10 |
| Linuron | 4.63 | 330-55-2 | 3.20 | 249 | 160 | 66 | 25 | 10 | 249 | 182 | 66 | 23 | 12 |
| Malaoxon | 2.78 | 1634-78-2 | 0.60 | 315 | 127 | 66 | 19 | 8 | 315 | 269 | 66 | 13 | 16 |
| Malathion | 4.42 | 121-75-5 | 2.36 | 331 | 127 | 86 | 17 | 8 | 331 | 285 | 86 | 11 | 16 |
| Mecarbam | 4.20 | 2595-54-2 | 2.29 | 330 | 227 | 76 | 15 | 12 | 330 | 97 | 76 | 45 | 6 |
| Mepanipyrim | 6.49 | 110235-47-7 | 3.28 | 224 | 106 | 119 | 35 | 6 | 224 | 77 | 119 | 49 | 8 |
| Metaflumizone | 9.16 | 139968-49-3 | 6.20 | 507 | 178 | 176 | 33 | 10 | 507 | 287 | 176 | 35 | 6 |
| Metalaxyl | 3.74 | 57837-19-1 | 1.65 | 280 | 220 | 81 | 19 | 12 | 280 | 160 | 81 | 31 | 10 |
| Metazachlor | 4.04 | 67129-08-2 | 2.13 | 278 | 134 | 66 | 29 | 8 | 278 | 210 | 66 | 15 | 10 |
| Methabenzthiazuron | 6.41 | 18691-97-9 | 2.64 | 222 | 165 | 76 | 23 | 10 | 222 | 150 | 76 | 45 | 12 |
| Methidathion | 5.85 | 950-37-8 | 2.20 | 303 | 145 | 79 | 13 | 26 | 303 | 85 | 79 | 29 | 14 |
| Methiocarb | 4.96 | 2032-65-7 | 2.92 | 226 | 169 | 74 | 15 | 10 | 226 | 169 | 74 | 25 | 8 |
| Methoprotryne | 3.49 | 841-06-5 | 2.82 | 272 | 198 | 61 | 31 | 8 | 272 | 240 | 61 | 27 | 14 |

| | | | | | | | | | | | | | |
|---|---|---|---|---|---|---|---|---|---|---|---|---|---|
| Methoxyfenozide | 4.81 | 161050-58-4 | 3.70 | 369 | 149 | 86 | 23 | 8 | 369 | 133 | 86 | 31 | 8 |
| Metobromuron | 3.67 | 3060-89-7 | 2.38 | 259 | 148 | 81 | 23 | 10 | 259 | 172 | 81 | 27 | 10 |
| Metolachlor | 4.50 | 51218-45-2 | 3.13 | 284 | 252 | 76 | 21 | 16 | 284 | 176 | 76 | 35 | 10 |
| Metrafenone | 7.43 | 220899-03-6 | 4.60 | 409 | 209 | 39 | 21 | 10 | 409 | 227 | 39 | 25 | 14 |
| Napropamid | 7.21 | 15299-99-7 | 3.36 | 272 | 129 | 96 | 21 | 16 | 272 | 199 | 96 | 19 | 12 |
| Norflurazon | 6.60 | 27314-13-2 | 2.30 | 304 | 284 | 99 | 31 | 16 | 304 | 160 | 99 | 41 | 8 |
| Oxadiargyl | 8.03 | 39807-15-3 | 3.95 | 341 | 230 | 56 | 21 | 14 | 341 | 151 | 56 | 24 | 15 |
| Oxycarboxim | 2.16 | 5259-88-1 | 0.77 | 268 | 175 | 96 | 21 | 16 | 268 | 147 | 96 | 29 | 16 |
| Paclobutrazol | 5.80 | 76738-62-0 | 3.20 | 294 | 70 | 84 | 39 | 10 | 294 | 125 | 84 | 49 | 6 |
| Pencycuron | 7.53 | 66063-05-6 | 4.82 | 329 | 125 | 196 | 35 | 14 | 329 | 99 | 196 | 83 | 6 |
| Penthiopyrad | 6.99 | 183675-82-3 | 4.00 | 360 | 276 | 141 | 21 | 26 | 360 | 177 | 141 | 41 | 12 |
| Pethoxamid | 6.14 | 106700-29-2 | 3.50 | 296 | 131 | 46 | 29 | 8 | 296 | 250 | 46 | 19 | 16 |
| Phenthoate | 5.87 | 2597-03-7 | 3.69 | 321 | 163 | 81 | 17 | 8 | 321 | 163 | 81 | 17 | 10 |
| Phosalone | 8.76 | 2310-17-0 | 4.38 | 368 | 182 | 69 | 19 | 14 | 368 | 11 | 69 | 51 | 8 |
| Phosmet | 5.36 | 732-11-6 | 2.78 | 318 | 133 | 79 | 51 | 8 | 318 | 160 | 79 | 19 | 14 |
| Phosphamidon | 3.21 | 13171-21-6 | 0.79 | 300 | 127 | 76 | 29 | 12 | 300 | 174 | 76 | 19 | 8 |
| Phoxim | 8.12 | 14816-18-3 | 4.39 | 299 | 129 | 46 | 17 | 6 | 299 | 77 | 46 | 41 | 12 |
| Picolinafen | 8.54 | 137641-05-5 | 4.90 | 377 | 238 | 76 | 43 | 14 | 377 | 145 | 76 | 69 | 10 |
| Picoxystrobin | 7.60 | 117428-22-5 | 3.60 | 368 | 205 | 81 | 15 | 10 | 368 | 145 | 81 | 27 | 10 |
| Pinoxaden | 7.11 | 243973-20-8 | 3.20 | 401 | 317 | 121 | 29 | 14 | 401 | 57 | 121 | 29 | 18 |
| Piperonyl Butoxyde | 9.61 | 51-03-6 | 4.75 | 356 | 177 | 64 | 17 | 14 | 356 | 119 | 64 | 49 | 8 |
| Pirimiphos-ethyl | 8.58 | 23505-41-1 | 4.85 | 334 | 198 | 81 | 29 | 10 | 334 | 182 | 81 | 27 | 12 |
| Pirimiphos-methyl | 7.29 | 29232-93-7 | 4.20 | 306 | 164 | 71 | 29 | 8 | 306 | 108 | 71 | 39 | 6 |
| Prochloraz | 7.77 | 67747-09-5 | 4.10 | 376 | 308 | 66 | 17 | 16 | 376 | 266 | 66 | 23 | 14 |
| Profenofos | 8.03 | 41198-08-7 | 4.68 | 373 | 303 | 76 | 25 | 18 | 373 | 97 | 76 | 43 | 16 |
| Prometon | 5.48 | 1610-18-0 | 2.99 | 226 | 142 | 41 | 29 | 10 | 226 | 184 | 41 | 23 | 10 |
| Prometryn | 5.67 | 7287-19-6 | 3.51 | 242 | 158 | 86 | 33 | 10 | 242 | 200 | 86 | 25 | 12 |
| Propaphos | 6.36 | 7292-16-2 | 3.67 | 305 | 221 | 90 | 21 | 14 | 305 | 141 | 90 | 35 | 8 |
| Propaquinazid | 9.89 | 189278-12-4 | 5.50 | 373 | 331 | 41 | 21 | 30 | 373 | 289 | 41 | 33 | 16 |
| Propargite | 9.71 | 2312-35-8 | 5.00 | 368 | 231 | 49 | 17 | 24 | 368 | 175 | 49 | 23 | 16 |
| Propazine | 4.87 | 139-40-2 | 2.93 | 230 | 146 | 96 | 33 | 12 | 230 | 188 | 96 | 23 | 12 |
| Propiconazole | 5.98 | 60207-90-1 | 3.72 | 342 | 159 | 61 | 37 | 8 | 342 | 69 | 61 | 33 | 4 |
| Propyzamide | 6.12 | 23950-58-5 | 3.43 | 256 | 173 | 76 | 31 | 8 | 256 | 190 | 76 | 19 | 12 |

| | | | | | | | | | | | | | |
|---|---|---|---|---|---|---|---|---|---|---|---|---|---|
| Prosulfocarb | 6.69 | 52888-80-9 | 4.65 | 252 | 91 | 71 | 31 | 16 | 252 | 128 | 71 | 19 | 8 |
| Pyraclostrobin | 7.99 | 175013-18-0 | 3.99 | 388 | 194 | 66 | 19 | 16 | 388 | 163 | 66 | 29 | 6 |
| Pyrazophos | 7.51 | 13457-18-6 | 3.80 | 374 | 222 | 86 | 29 | 12 | 374 | 194 | 86 | 43 | 10 |
| Pyridaben | 9.64 | 96489-71-3 | 6.37 | 365 | 309 | 96 | 19 | 18 | 365 | 147 | 96 | 31 | 8 |
| Pyridafenthion | 8.72 | 119-12-0 | 3.20 | 341 | 189 | 61 | 29 | 10 | 341 | 205 | 61 | 27 | 10 |
| Pyridate | 10.09 | 55512-33-9 | 6.60 | 379 | 207 | 56 | 25 | 14 | 379 | 351 | 56 | 15 | 16 |
| Rimsulfuron | 2.45 | 122931-48-0 | -1.46 | 432 | 182 | 161 | 29 | 16 | 432 | 325 | 161 | 21 | 14 |
| Saflufenacil | 5.14 | 372137-35-4 | 2.60 | 501 | 198 | 156 | 61 | 20 | 501 | 349 | 156 | 41 | 10 |
| Spinosyn-A | 5.01 | 131929-60-7 | 2.80 | 733 | 142 | 84 | 39 | 8 | 98 | 84 | 84 | 107 | 28 |
| Spirotetramat | 4.97 | 203313-25-1 | 2.51 | 374 | 216 | 116 | 45 | 14 | 374 | 302 | 116 | 23 | 14 |
| Spiroxamine | 5.05 | 118134-30-8 | 2.89 | 298 | 144 | 51 | 29 | 10 | 298 | 100 | 51 | 49 | 6 |
| Sulfotep | 7.06 | 3689-24-5 | 3.99 | 323 | 97 | 96 | 45 | 4 | 323 | 115 | 96 | 39 | 8 |
| Tebufenozide | 7.98 | 112410-23-8 | 4.25 | 353 | 133 | 71 | 23 | 6 | 353 | 297 | 71 | 15 | 16 |
| Tebufenpyrad | 8.23 | 119168-77-3 | 4.93 | 334 | 145 | 86 | 37 | 8 | 334 | 117 | 86 | 47 | 12 |
| Tepraloxydim | 6.65 | 149979-41-9 | 1.50 | 342 | 250 | 116 | 19 | 26 | 242 | 166 | 116 | 29 | 18 |
| Terbufos | 9.75 | 13071-79-9 | 4.48 | 289 | 103 | 69 | 13 | 6 | 289 | 233 | 69 | 9 | 14 |
| Thiobencarb | 9.35 | 28249-77-6 | 4.23 | 258 | 125 | 53 | 29 | 12 | 258 | 89 | 53 | 69 | 14 |
| Tolfenpyrad | 9.84 | 129558-76-5 | 5.61 | 384 | 197 | 121 | 41 | 16 | 384 | 154 | 121 | 59 | 10 |
| Tralkoxydim | 5.81 | 87820-88-0 | 2.10 | 330 | 284 | 126 | 17 | 18 | 330 | 138 | 126 | 27 | 10 |
| Triadimefon | 6.52 | 43121-43-3 | 2.77 | 294 | 197 | 71 | 23 | 14 | 294 | 69 | 71 | 59 | 8 |
| Triallate | 9.71 | 2303-17-5 | 4.60 | 304 | 86 | 81 | 25 | 14 | 304 | 143 | 81 | 37 | 12 |
| Tridemorph | 10.01 | 24602-86-6 | 6.90 | 298 | 130 | 51 | 35 | 6 | 298 | 116 | 51 | 33 | 8 |
| Triforine | 6.08 | 26644-46-2 | 2.20 | 435 | 390 | 81 | 15 | 10 | 435 | 98 | 81 | 31 | 10 |
| Zoxamide | 8.87 | 156052-68-5 | 3.76 | 336 | 159 | 100 | 47 | 16 | 336 | 187 | 100 | 29 | 14 |

Table S2: Pesticide recoveries and the relative standard deviations using different purification sorbents, freezing process and without clean-up for QuEChERS purification step of rapeseed extracts.

| Analytes | Without cleanup | | | | Freezing | | | | C18/PSA | | | | EMR-Lipid | | | | Z-Sep | | | | Z-Sep+ | | | |
|---|---|---|---|---|---|---|---|---|---|---|---|---|---|---|---|---|---|---|---|---|---|---|---|---|
| | 10 µg/kg | | 50 µg/kg | | 10 µg/kg | | 50 µg/kg | | 10 µg/kg | | 50 µg/kg | | 10 µg/kg | | 50 µg/kg | | 10 µg/kg | | 50 µg/kg | | 10 µg/kg | | 50 µg/kg | |
| | Rec, % | RSD, % | Rec, % | RSD, % | Rec, % | RSD, % | Rec, % | RSD, % | Rec, % | RSD, % | Rec, % | RSD, % | Rec, % | RSD, % | Rec, % | RSD, % | Rec, % | RSD, % | Rec, % | RSD, % | Rec, % | RSD, % | Rec, % | RSD, % |
| Acephate | 63 | 11 | 64 | 7 | 66 | 8 | 58 | 3 | 72 | 4 | 68 | 22 | 80 | 12 | 82 | 4 | 59 | 8 | 60 | 9 | 81 | 12 | 67 | 4 |
| Acetochlor | 44 | 17 | 54 | 16 | 61 | 8 | 60 | 2 | 61 | 13 | 60 | 5 | 80 | 9 | 71 | 2 | 53 | 5 | 50 | 5 | 64 | 14 | 63 | 6 |
| Acibenzolar-S-methyl | 88 | 8 | 118 | 20 | 53 | 13 | 50 | 5 | 54 | 12 | 34 | 4 | 50 | 9 | 58 | 11 | 34 | 12 | 41 | 17 | 0 | / | 58 | 14 |
| Alachlor | 62 | 11 | 66 | 9 | 66 | 8 | 63 | 2 | 61 | 5 | 56 | 3 | 82 | 5 | 80 | 6 | 54 | 3 | 52 | 7 | 56 | 18 | 48 | 3 |
| Aldicarb sulfone | 92 | 3 | 89 | 4 | 45 | 6 | 31 | 11 | 90 | 11 | 82 | 6 | 103 | 4 | 101 | 7 | 0 | / | 0 | / | 104 | 11 | 91 | 5 |
| Ametoctradin | 82 | 19 | 94 | 10 | 77 | 14 | 65 | 7 | 56 | 10 | 59 | 6 | 77 | 4 | 79 | 7 | 65 | 11 | 59 | 7 | 57 | 18 | 46 | 11 |
| Ametryn | 48 | 7 | 52 | 8 | 41 | 5 | 40 | 2 | 29 | 4 | 28 | 5 | 64 | 5 | 63 | 8 | 34 | 2 | 33 | 6 | 49 | 22 | 47 | 9 |
| Anilofos | 64 | 14 | 44 | 3 | 51 | 7 | 42 | 3 | 37 | 6 | 28 | 3 | 67 | 14 | 57 | 3 | 41 | 4 | 33 | 8 | 72 | 19 | 51 | 4 |
| Azaconazole | 89 | 6 | 99 | 8 | 56 | 11 | 53 | 3 | 35 | 5 | 39 | 6 | 66 | 6 | 70 | 8 | 46 | 5 | 46 | 4 | 60 | 17 | 58 | 8 |
| Azinphos-methyl | 98 | 3 | 56 | 6 | 51 | 20 | 45 | 4 | 0 | / | 26 | 19 | 72 | 12 | 61 | 9 | 40 | 10 | 36 | 5 | 58 | 7 | 64 | 6 |
| Aziprotryne | 73 | 12 | 77 | 10 | 79 | 13 | 68 | 6 | 62 | 5 | 67 | 1 | 80 | 8 | 78 | 6 | 62 | 6 | 55 | 7 | 66 | 22 | 62 | 9 |
| Azoxystrobin | 74 | 9 | 72 | 5 | 63 | 10 | 56 | 3 | 47 | 8 | 44 | 5 | 75 | 6 | 76 | 5 | 51 | 2 | 46 | 6 | 59 | 19 | 55 | 9 |
| Barban | 109 | 18 | 90 | 21 | 73 | 19 | 67 | 10 | 74 | 12 | 59 | 11 | 52 | 36 | 70 | 17 | 74 | 3 | 60 | 10 | 0 | / | 60 | 31 |
| Benalaxyl | 64 | 13 | 60 | 14 | 72 | 17 | 65 | 2 | 57 | 4 | 55 | 3 | 90 | 5 | 91 | 9 | 63 | 5 | 60 | 11 | 69 | 19 | 71 | 14 |
| Benoxacor | 62 | 8 | 55 | 8 | 65 | 6 | 56 | 6 | 41 | 10 | 42 | 10 | 74 | 6 | 71 | 3 | 49 | 7 | 47 | 9 | 63 | 18 | 53 | 13 |
| Bifenox | 68 | 18 | 72 | 15 | 54 | 13 | 61 | 2 | 29 | 28 | 34 | 18 | 49 | 19 | 63 | 13 | 47 | 19 | 54 | 7 | 51 | 12 | 48 | 25 |
| Bixafen | 64 | 10 | 60 | 7 | 62 | 12 | 66 | 5 | 57 | 13 | 53 | 13 | 71 | 7 | 78 | 7 | 44 | 15 | 52 | 11 | 69 | 13 | 68 | 8 |
| Boscalid | 123 | 15 | 90 | 13 | 98 | 4 | 69 | 6 | 37 | 13 | 39 | 8 | 87 | 9 | 72 | 6 | 89 | 14 | 51 | 9 | 114 | 18 | 76 | 5 |
| Bupirimate | 68 | 12 | 60 | 13 | 63 | 9 | 61 | 3 | 50 | 4 | 48 | 5 | 73 | 5 | 71 | 4 | 52 | 5 | 52 | 5 | 62 | 25 | 62 | 5 |
| Buprofezin | 58 | 6 | 65 | 6 | 68 | 4 | 67 | 4 | 62 | 4 | 60 | 2 | 74 | 7 | 76 | 4 | 63 | 5 | 62 | 5 | 62 | 18 | 61 | 6 |
| Chlorfenvinphos | 66 | 19 | 70 | 9 | 57 | 5 | 53 | 3 | 40 | 7 | 44 | 4 | 66 | 7 | 71 | 7 | 45 | 5 | 45 | 4 | 72 | 12 | 69 | 7 |
| Chlorobromuron | 61 | 13 | 74 | 8 | 53 | 13 | 58 | 1 | 48 | 4 | 45 | 4 | 55 | 9 | 54 | 3 | 47 | 4 | 46 | 4 | 58 | 22 | 49 | 7 |
| Chlorfluazuron | 72 | 8 | 82 | 11 | 67 | 15 | 72 | 1 | 48 | 12 | 59 | 7 | 44 | 11 | 42 | 2 | 58 | 5 | 70 | 7 | 82 | 18 | 77 | 5 |

| | | | | | | | | | | | | | | | | | | | | | | | | |
|---|---|---|---|---|---|---|---|---|---|---|---|---|---|---|---|---|---|---|---|---|---|---|---|---|
| Chlorpyrifos | 59 | 6 | 61 | 10 | 59 | 6 | 57 | 5 | 54 | 4 | 45 | 7 | 76 | 7 | 72 | 2 | 53 | 2 | 54 | 5 | 59 | 14 | 57 | 7 |
| Chlorpyrifos-methyl | 64 | 22 | 58 | 15 | 57 | 15 | 76 | 9 | 54 | 14 | 60 | 7 | 75 | 11 | 72 | 6 | 59 | 19 | 70 | 8 | 47 | 19 | 70 | 9 |
| Cinosulfuron | 50 | 9 | 60 | 4 | 28 | 4 | 29 | 4 | 16 | 5 | 22 | 5 | 114 | 4 | 118 | 4 | 34 | 5 | 35 | 6 | 80 | 11 | 79 | 6 |
| Climbazole | 64 | 7 | 56 | 9 | 49 | 7 | 48 | 3 | 29 | 6 | 35 | 7 | 81 | 10 | 74 | 5 | 37 | 7 | 39 | 6 | 64 | 29 | 57 | 6 |
| Clodinafop-propargyl | 71 | 13 | 77 | 12 | 62 | 7 | 63 | 3 | 42 | 7 | 44 | 6 | 79 | 8 | 92 | 3 | 49 | 6 | 52 | 5 | 75 | 18 | 79 | 7 |
| Clofentezine | 61 | 10 | 70 | 4 | 72 | 13 | 76 | 3 | 54 | 3 | 51 | 5 | 45 | 11 | 53 | 8 | 58 | 2 | 63 | 10 | 49 | 13 | 53 | 16 |
| Cloquintocet-mexyl | 66 | 1 | 70 | 8 | 71 | 10 | 70 | 2 | 62 | 4 | 62 | 2 | 74 | 4 | 76 | 3 | 63 | 3 | 63 | 6 | 59 | 21 | 56 | 4 |
| Coumaphos | 61 | 9 | 83 | 11 | 59 | 8 | 57 | 2 | 37 | 11 | 45 | 2 | 66 | 9 | 72 | 6 | 51 | 7 | 48 | 3 | 75 | 11 | 79 | 6 |
| Coumatetralyl | 45 | 6 | 48 | 11 | 53 | 8 | 49 | 6 | 46 | 3 | 46 | 2 | 70 | 5 | 67 | 6 | 36 | 6 | 34 | 4 | 54 | 27 | 50 | 6 |
| Crufomate | 65 | 10 | 75 | 9 | 83 | 12 | 81 | 3 | 63 | 13 | 63 | 2 | 91 | 11 | 95 | 7 | 67 | 3 | 69 | 6 | 63 | 27 | 59 | 6 |
| Cyanazine | 49 | 8 | 56 | 5 | 77 | 6 | 72 | 4 | 28 | 19 | 37 | 4 | 80 | 5 | 98 | 3 | 67 | 4 | 58 | 4 | 61 | 12 | 66 | 5 |
| Cycloxydim | 61 | 5 | 61 | 9 | 57 | 15 | 53 | 5 | 55 | 7 | 54 | 2 | 67 | 5 | 68 | 6 | 48 | 4 | 50 | 5 | 52 | 13 | 50 | 7 |
| Cyflumetofen | 76 | 20 | 77 | 4 | 85 | 12 | 75 | 5 | 82 | 22 | 56 | 11 | 100 | 11 | 87 | 4 | 81 | 2 | 76 | 7 | 85 | 26 | 77 | 8 |
| Cymiazole | 37 | 8 | 36 | 3 | 40 | 7 | 38 | 3 | 42 | 5 | 38 | 2 | 50 | 4 | 44 | 2 | 33 | 6 | 30 | 5 | 45 | 7 | 38 | 2 |
| Cyproconazole | 85 | 5 | 81 | 11 | 59 | 10 | 53 | 3 | 64 | 6 | 53 | 9 | 83 | 6 | 85 | 5 | 57 | 5 | 51 | 5 | 63 | 17 | 49 | 8 |
| Cyprodinil | 63 | 5 | 29 | 1 | 68 | 18 | 60 | 4 | 42 | 6 | 45 | 2 | 116 | 20 | 29 | 13 | 57 | 8 | 52 | 7 | 0 | / | 44 | 7 |
| Demeton-S-methyl sulfone | 48 | 2 | 49 | 5 | 61 | 7 | 59 | 1 | 50 | 4 | 51 | 3 | 59 | 2 | 57 | 4 | 57 | 2 | 57 | 5 | 57 | 10 | 51 | 7 |
| Desmetryn | 45 | 3 | 49 | 8 | 41 | 5 | 39 | 4 | 13 | 8 | 17 | 4 | 61 | 4 | 68 | 2 | 32 | 2 | 29 | 6 | 48 | 12 | 38 | 6 |
| Diafenthiuron | 73 | 6 | 79 | 11 | 57 | 18 | 56 | 3 | 95 | 12 | 96 | 3 | 70 | 4 | 74 | 2 | 45 | 2 | 45 | 7 | 63 | 18 | 50 | 5 |
| Dialifos | 58 | 17 | 64 | 11 | 52 | 15 | 61 | 7 | 34 | 22 | 49 | 7 | 57 | 7 | 63 | 8 | 48 | 11 | 52 | 6 | 64 | 12 | 60 | 8 |
| Diazinon | 57 | 11 | 54 | 11 | 71 | 9 | 70 | 7 | 57 | 4 | 58 | 12 | 72 | 1 | 60 | 4 | 61 | 2 | 62 | 6 | 76 | 13 | 73 | 7 |
| Diclofop-methyl | 63 | 15 | 62 | 7 | 57 | 13 | 61 | 5 | 42 | 16 | 45 | 6 | 63 | 8 | 61 | 4 | 48 | 8 | 57 | 7 | 67 | 20 | 61 | 6 |
| Diethofencarb | 60 | 4 | 62 | 7 | 58 | 8 | 55 | 5 | 40 | 8 | 43 | 4 | 86 | 1 | 95 | 4 | 46 | 8 | 44 | 8 | 59 | 14 | 51 | 5 |
| Difenoconazole | 79 | 13 | 86 | 6 | 76 | 13 | 72 | 3 | 81 | 14 | 61 | 5 | 86 | 11 | 97 | 6 | 68 | 3 | 66 | 5 | 79 | 17 | 66 | 6 |
| Diflufenican | 70 | 8 | 74 | 7 | 70 | 8 | 62 | 7 | 43 | 10 | 51 | 20 | 58 | 9 | 75 | 10 | 63 | 4 | 56 | 16 | 71 | 23 | 81 | 13 |
| Dimethachlor | 58 | 11 | 61 | 12 | 61 | 7 | 61 | 5 | 42 | 3 | 49 | 6 | 68 | 5 | 64 | 4 | 48 | 2 | 50 | 6 | 71 | 31 | 70 | 9 |
| Dimethenamid | 58 | 15 | 65 | 16 | 59 | 9 | 59 | 4 | 50 | 8 | 43 | 1 | 70 | 9 | 73 | 5 | 46 | 5 | 48 | 6 | 59 | 20 | 60 | 9 |
| Dimethomorph | 83 | 12 | 86 | 6 | 62 | 9 | 57 | 2 | 31 | 7 | 37 | 6 | 81 | 5 | 88 | 9 | 50 | 8 | 46 | 5 | 73 | 21 | 72 | 1 |
| Dimoxystrobin | 66 | 13 | 70 | 9 | 68 | 11 | 66 | 8 | 46 | 28 | 42 | 4 | 76 | 14 | 84 | 8 | 52 | 8 | 55 | 5 | 72 | 21 | 74 | 6 |

| | | | | | | | | | | | | | | | | | | | | | | | | | | | | | | | | | | | | | | | | | | | | | | |
|---|---|---|---|---|---|---|---|---|---|---|---|---|---|---|---|---|---|---|---|---|
| Dioxathion | 72 | 7 | 78 | 6 | 70 | 10 | 71 | 4 | 54 | 4 | 60 | 3 | 69 | 7 | 73 | 4 | 60 | 5 | 64 | 5 | 69 | 21 | 57 | 5 |
| Dipropetryn | 45 | 8 | 56 | 13 | 57 | 3 | 52 | 2 | 55 | 9 | 52 | 6 | 86 | 9 | 80 | 3 | 51 | 4 | 47 | 2 | 57 | 14 | 47 | 3 |
| Ditalimphos | 49 | 14 | 54 | 7 | 54 | 6 | 46 | 3 | 47 | 7 | 47 | 4 | 72 | 7 | 78 | 5 | 50 | 5 | 47 | 5 | 58 | 17 | 54 | 7 |
| Emamectin Ba | 85 | 6 | 94 | 9 | 77 | 2 | 74 | 4 | 102 | 9 | 97 | 6 | 92 | 6 | 93 | 3 | 68 | 9 | 67 | 6 | 101 | 18 | 94 | 6 |
| Emamectin Bb | 72 | 9 | 71 | 9 | 93 | 14 | 73 | 4 | 100 | 6 | 103 | 4 | 89 | 4 | 86 | 3 | 75 | 10 | 61 | 5 | 102 | 18 | 87 | 9 |
| Epoxiconazole | 59 | 15 | 61 | 17 | 72 | 13 | 72 | 9 | 57 | 10 | 53 | 6 | 104 | 7 | 83 | 5 | 62 | 18 | 55 | 10 | 66 | 16 | 61 | 6 |
| Etaconazole | 78 | 21 | 78 | 18 | 55 | 20 | 55 | 5 | 46 | 29 | 50 | 12 | 117 | 11 | 96 | 8 | 65 | 17 | 53 | 7 | 0 | / | 51 | 14 |
| Ethion | 56 | 4 | 61 | 6 | 64 | 5 | 63 | 3 | 61 | 5 | 59 | 3 | 61 | 7 | 58 | 4 | 54 | 2 | 56 | 5 | 71 | 15 | 62 | 7 |
| Ethofumesate | 64 | 14 | 72 | 9 | 0 | / | 87 | 15 | 53 | 17 | 52 | 4 | 72 | 13 | 85 | 4 | 0 | / | 0 | / | 75 | 14 | 55 | 11 |
| Ethoprophos | 48 | 17 | 63 | 9 | 63 | 6 | 58 | 3 | 57 | 8 | 53 | 9 | 83 | 6 | 87 | 7 | 52 | 6 | 49 | 5 | 60 | 20 | 52 | 12 |
| Etoxazole | 61 | 7 | 64 | 10 | 59 | 11 | 60 | 4 | 54 | 4 | 56 | 4 | 40 | 11 | 29 | 8 | 50 | 2 | 50 | 5 | 68 | 22 | 61 | 5 |
| Etrimfos | 63 | 15 | 55 | 19 | 67 | 10 | 71 | 5 | 62 | 10 | 64 | 10 | 74 | 3 | 72 | 5 | 61 | 14 | 66 | 7 | 68 | 21 | 64 | 6 |
| Famoxadone | 70 | 18 | 77 | 17 | 73 | 12 | 74 | 13 | 47 | 7 | 60 | 12 | 87 | 13 | 86 | 5 | 64 | 6 | 63 | 6 | 78 | 33 | 73 | 4 |
| Fenamiphos | 54 | 14 | 75 | 15 | 76 | 7 | 73 | 5 | 54 | 9 | 62 | 5 | 80 | 7 | 80 | 2 | 59 | 3 | 61 | 2 | 61 | 20 | 48 | 5 |
| Fenarimol | 69 | 15 | 77 | 16 | 74 | 14 | 65 | 2 | 74 | 5 | 63 | 4 | 95 | 14 | 92 | 5 | 53 | 19 | 59 | 5 | 49 | 46 | 91 | 7 |
| Fenazaquin | 62 | 6 | 54 | 9 | 58 | 9 | 54 | 2 | 48 | 16 | 43 | 8 | 58 | 5 | 37 | 2 | 53 | 2 | 49 | 6 | 56 | 19 | 41 | 5 |
| Fenbuconazole | 76 | 14 | 83 | 18 | 71 | 12 | 63 | 6 | 47 | 10 | 46 | 5 | 79 | 8 | 84 | 8 | 57 | 9 | 58 | 7 | 70 | 28 | 74 | 14 |
| Fenpropidin | 72 | 8 | 74 | 8 | 60 | 2 | 54 | 3 | 50 | 5 | 46 | 4 | 89 | 8 | 92 | 4 | 60 | 6 | 52 | 3 | 73 | 22 | 63 | 7 |
| Fenpropimorph | 61 | 7 | 84 | 8 | 60 | 8 | 52 | 5 | 34 | 6 | 46 | 12 | 87 | 5 | 89 | 4 | 50 | 2 | 46 | 4 | 49 | 15 | 51 | 7 |
| Fenpyroximate | 72 | 7 | 73 | 7 | 63 | 19 | 66 | 5 | 53 | 5 | 57 | 1 | 52 | 5 | 51 | 4 | 55 | 2 | 60 | 5 | 75 | 24 | 71 | 5 |
| Fensulfothion | 53 | 4 | 63 | 8 | 49 | 4 | 43 | 2 | 28 | 7 | 30 | 3 | 61 | 5 | 85 | 9 | 40 | 1 | 37 | 7 | 59 | 27 | 54 | 8 |
| Fensulfothion-oxon | 33 | 5 | 31 | 5 | 34 | 5 | 29 | 2 | 20 | 5 | 20 | 4 | 77 | 6 | 75 | 6 | 28 | 3 | 22 | 5 | 39 | 8 | 29 | 4 |
| Fensulfothion-sulfone | 91 | 7 | 97 | 8 | 50 | 5 | 48 | 6 | 12 | 17 | 28 | 5 | 76 | 3 | 78 | 5 | 40 | 5 | 40 | 5 | 66 | 21 | 65 | 5 |
| Fenthion | 59 | 13 | 61 | 9 | 67 | 8 | 71 | 7 | 62 | 8 | 60 | 7 | 70 | 5 | 70 | 5 | 61 | 7 | 64 | 5 | 61 | 22 | 55 | 10 |
| Fenthion sulfone | 44 | 16 | 41 | 13 | 30 | 17 | 48 | 15 | 23 | 5 | 27 | 3 | 62 | 16 | 81 | 4 | 24 | 17 | 38 | 11 | 64 | 19 | 56 | 6 |
| Fenthion sulfoxide | 43 | 2 | 37 | 7 | 38 | 2 | 35 | 4 | 24 | 9 | 26 | 5 | 49 | 2 | 45 | 4 | 35 | 5 | 29 | 7 | 51 | 20 | 43 | 5 |
| Flamprop-isopropyl | 59 | 6 | 58 | 13 | 62 | 9 | 60 | 5 | 47 | 5 | 46 | 5 | 73 | 7 | 82 | 9 | 49 | 6 | 50 | 7 | 74 | 13 | 69 | 18 |
| Flazasulfuron | 102 | 12 | 125 | 13 | 32 | 7 | 32 | 4 | 20 | 16 | 23 | 11 | 127 | 7 | 142 | 3 | 41 | 5 | 41 | 7 | 100 | 35 | 110 | 4 |
| Florasulam | 57 | 5 | 63 | 8 | 57 | 6 | 58 | 7 | 21 | 14 | 25 | 7 | 88 | 5 | 98 | 5 | 74 | 7 | 46 | 4 | 92 | 15 | 90 | 9 |
| Flubendiamide | 69 | 19 | 79 | 16 | 61 | 19 | 70 | 9 | 78 | 7 | 49 | 4 | 79 | 15 | 116 | 16 | 57 | 18 | 59 | 4 | 77 | 23 | 95 | 13 |

| | | | | | | | | | | | | | | | | | | | | | | | | |
|---|---|---|---|---|---|---|---|---|---|---|---|---|---|---|---|---|---|---|---|---|---|---|---|---|
| Flucycloxuron | 80 | 7 | 86 | 11 | 79 | 17 | 79 | 6 | 47 | 15 | 69 | 3 | 64 | 4 | 63 | 6 | 65 | 7 | 71 | 7 | 82 | 21 | 79 | 4 |
| Flufenacet | 66 | 14 | 75 | 9 | 90 | 14 | 79 | 12 | 49 | 4 | 57 | 30 | 72 | 7 | 73 | 4 | 72 | 2 | 68 | 7 | 105 | 27 | 103 | 4 |
| Flufenoxuron | 71 | 4 | 68 | 8 | 70 | 29 | 79 | 4 | 59 | 15 | 62 | 6 | 54 | 6 | 53 | 3 | 54 | 7 | 70 | 10 | 77 | 26 | 74 | 4 |
| Fluopicolide | 64 | 8 | 76 | 10 | 52 | 14 | 46 | 14 | 32 | 9 | 32 | 13 | 51 | 5 | 71 | 5 | 41 | 6 | 35 | 6 | 64 | 17 | 52 | 10 |
| Flurochloridon | 76 | 8 | 86 | 2 | 55 | 16 | 57 | 4 | 36 | 11 | 37 | 6 | 66 | 5 | 56 | 10 | 44 | 10 | 49 | 3 | 57 | 21 | 50 | 12 |
| Flurprimidol | 82 | 20 | 80 | 6 | 71 | 13 | 64 | 6 | 59 | 6 | 54 | 8 | 85 | 7 | 86 | 4 | 60 | 8 | 57 | 2 | 70 | 44 | 59 | 12 |
| Flurtamone | 65 | 7 | 63 | 6 | 55 | 8 | 52 | 2 | 36 | 5 | 35 | 7 | 84 | 4 | 77 | 6 | 43 | 3 | 42 | 5 | 62 | 19 | 50 | 4 |
| Flusilazole | 64 | 16 | 75 | 10 | 80 | 8 | 75 | 4 | 66 | 10 | 64 | 8 | 78 | 8 | 68 | 6 | 64 | 3 | 61 | 7 | 61 | 14 | 54 | 6 |
| Fluxapyroxad | 70 | 6 | 79 | 11 | 57 | 11 | 55 | 4 | 28 | 4 | 32 | 6 | 56 | 12 | 87 | 9 | 38 | 9 | 44 | 6 | 68 | 18 | 61 | 12 |
| Fluxastrobin | 40 | 30 | 73 | 9 | 52 | 8 | 51 | 10 | 51 | 5 | 45 | 5 | 73 | 6 | 86 | 9 | 35 | 10 | 40 | 6 | 57 | 24 | 59 | 12 |
| Fonofos | 68 | 13 | 55 | 19 | 88 | 13 | 68 | 9 | 74 | 9 | 58 | 6 | 81 | 15 | 83 | 8 | 82 | 9 | 70 | 9 | 89 | 18 | 58 | 8 |
| Foramsulfuron | 93 | 7 | 107 | 8 | 17 | 10 | 17 | 2 | 14 | 8 | 14 | 3 | 145 | 4 | 145 | 5 | 42 | 4 | 39 | 3 | 85 | 20 | 66 | 8 |
| Forchlofenuron | 67 | 10 | 58 | 5 | 71 | 3 | 67 | 3 | 0 | / | 46 | 15 | 63 | 8 | 82 | 2 | 60 | 7 | 57 | 5 | 93 | 25 | 77 | 11 |
| Furmecyclox | 50 | 5 | 51 | 13 | 68 | 5 | 65 | 3 | 52 | 5 | 68 | 6 | 65 | 6 | 60 | 10 | 59 | 2 | 55 | 5 | 55 | 10 | 49 | 7 |
| Haloxyfop-methyl | 70 | 6 | 71 | 7 | 69 | 11 | 70 | 4 | 43 | 6 | 47 | 2 | 76 | 6 | 78 | 5 | 58 | 3 | 61 | 4 | 75 | 16 | 77 | 10 |
| Heptenophos | 102 | 14 | 58 | 9 | 60 | 13 | 62 | 8 | 47 | 5 | 47 | 8 | 77 | 7 | 61 | 7 | 47 | 18 | 47 | 5 | 71 | 27 | 55 | 7 |
| Hexazinone | 37 | 5 | 37 | 6 | 34 | 4 | 32 | 3 | 21 | 3 | 20 | 4 | 46 | 5 | 50 | 4 | 30 | 4 | 26 | 6 | 42 | 13 | 35 | 6 |
| Hexythiazox | 52 | 6 | 57 | 10 | 57 | 10 | 59 | 6 | 37 | 14 | 47 | 4 | 45 | 12 | 46 | 7 | 49 | 11 | 51 | 8 | 56 | 14 | 54 | 3 |
| Imazalil | 47 | 5 | 45 | 6 | 55 | 7 | 52 | 5 | 44 | 8 | 43 | 7 | 82 | 5 | 75 | 7 | 45 | 8 | 41 | 6 | 69 | 12 | 62 | 8 |
| Imazosulfuron | 107 | 17 | 116 | 11 | 55 | 7 | 48 | 12 | 37 | 9 | 32 | 2 | 107 | 10 | 103 | 4 | 82 | 8 | 54 | 8 | 131 | 24 | 118 | 5 |
| Imibenconazole | 61 | 1 | 71 | 3 | 112 | 11 | 78 | 7 | 58 | 13 | 61 | 3 | 56 | 6 | 52 | 9 | 86 | 12 | 68 | 12 | 65 | 19 | 64 | 3 |
| Indoxacarb | 103 | 12 | 102 | 9 | 91 | 15 | 85 | 5 | 46 | 11 | 77 | 8 | 106 | 3 | 108 | 9 | 88 | 7 | 80 | 8 | 117 | 20 | 83 | 2 |
| Ipconazole | 80 | 7 | 81 | 5 | 71 | 18 | 74 | 6 | 52 | 10 | 63 | 6 | 116 | 4 | 117 | 7 | 105 | 8 | 81 | 8 | 72 | 23 | 67 | 6 |
| Isofenphos | 61 | 6 | 61 | 5 | 91 | 11 | 80 | 6 | 40 | 9 | 46 | 3 | 77 | 5 | 81 | 9 | 83 | 4 | 74 | 8 | 64 | 16 | 62 | 4 |
| Isofenphos-methyl | 72 | 11 | 75 | 12 | 58 | 6 | 53 | 4 | 35 | 15 | 36 | 8 | 72 | 4 | 63 | 6 | 53 | 4 | 47 | 4 | 73 | 16 | 72 | 7 |
| Isopyrazam | 67 | 9 | 73 | 8 | 70 | 10 | 65 | 2 | 54 | 9 | 55 | 5 | 78 | 8 | 83 | 6 | 62 | 5 | 59 | 4 | 80 | 16 | 72 | 3 |
| Isoxaflutole | 104 | 5 | 94 | 6 | 73 | 7 | 64 | 6 | 63 | 4 | 44 | 4 | 168 | 7 | 143 | 3 | 79 | 8 | 69 | 6 | 112 | 21 | 99 | 6 |
| Kresoxim-methyl | 70 | 16 | 67 | 11 | 72 | 6 | 66 | 4 | 58 | 15 | 50 | 8 | 84 | 10 | 71 | 5 | 53 | 10 | 56 | 4 | 66 | 14 | 64 | 4 |
| Linuron | 51 | 6 | 54 | 11 | 61 | 13 | 56 | 3 | 46 | 8 | 48 | 6 | 66 | 6 | 58 | 7 | 46 | 11 | 43 | 5 | 75 | 10 | 54 | 6 |
| Malaoxon | 37 | 3 | 41 | 7 | 49 | 6 | 36 | 2 | 36 | 1 | 23 | 3 | 64 | 6 | 59 | 3 | 45 | 3 | 31 | 7 | 25 | 17 | 45 | 6 |

| | | | | | | | | | | | | | | | | | | | | | | | | |
|---|---|---|---|---|---|---|---|---|---|---|---|---|---|---|---|---|---|---|---|---|---|---|---|---|
| Malathion | 64 | 8 | 81 | 9 | 68 | 8 | 64 | 3 | 43 | 9 | 48 | 2 | 75 | 3 | 76 | 7 | 58 | 4 | 54 | 3 | 68 | 21 | 60 | 3 |
| Mecarbam | 64 | 6 | 51 | 4 | 48 | 8 | 46 | 4 | 38 | 8 | 37 | 4 | 74 | 10 | 73 | 1 | 39 | 10 | 37 | 5 | 64 | 14 | 66 | 11 |
| Mepanipyrim | 50 | 11 | 49 | 8 | 72 | 7 | 54 | 2 | 52 | 3 | 49 | 5 | 66 | 5 | 81 | 9 | 57 | 4 | 46 | 8 | 47 | 11 | 44 | 6 |
| Metaflumizone | 86 | 10 | 86 | 17 | 79 | 15 | 83 | 5 | 65 | 22 | 75 | 9 | 89 | 10 | 68 | 11 | 69 | 10 | 74 | 8 | 114 | 17 | 94 | 14 |
| Metalaxyl | 65 | 5 | 59 | 3 | 63 | 7 | 55 | 3 | 41 | 9 | 41 | 7 | 95 | 5 | 92 | 7 | 53 | 2 | 44 | 5 | 80 | 24 | 65 | 5 |
| Metazachlor | 38 | 16 | 56 | 7 | 49 | 7 | 53 | 4 | 34 | 6 | 36 | 5 | 67 | 5 | 67 | 4 | 42 | 4 | 43 | 4 | 57 | 14 | 44 | 8 |
| Methabenzthiazuron | 48 | 8 | 55 | 7 | 59 | 7 | 51 | 2 | 42 | 6 | 42 | 3 | 55 | 5 | 64 | 5 | 45 | 4 | 40 | 6 | 59 | 20 | 55 | 3 |
| Methidathion | 38 | 18 | 60 | 4 | 50 | 15 | 57 | 6 | 45 | 5 | 42 | 8 | 45 | 6 | 60 | 5 | 40 | 4 | 49 | 4 | 49 | 21 | 44 | 7 |
| Methiocarb | 56 | 3 | 68 | 10 | 64 | 10 | 61 | 4 | 59 | 7 | 60 | 3 | 65 | 2 | 72 | 3 | 49 | 4 | 48 | 4 | 87 | 9 | 63 | 5 |
| Methoprotryne | 51 | 6 | 57 | 6 | 58 | 10 | 55 | 6 | 43 | 4 | 46 | 7 | 81 | 4 | 84 | 7 | 49 | 5 | 47 | 6 | 60 | 13 | 58 | 6 |
| Methoxyfenozide | 53 | 18 | 72 | 12 | 50 | 9 | 50 | 4 | 37 | 6 | 31 | 5 | 54 | 13 | 72 | 7 | 38 | 5 | 42 | 7 | 61 | 14 | 58 | 6 |
| Metobromuron | 55 | 7 | 45 | 9 | 43 | 5 | 38 | 6 | 31 | 8 | 33 | 4 | 65 | 9 | 67 | 7 | 37 | 6 | 32 | 6 | 60 | 9 | 36 | 5 |
| Metolachlor | 52 | 13 | 59 | 10 | 62 | 6 | 60 | 3 | 46 | 1 | 50 | 3 | 78 | 6 | 78 | 3 | 50 | 8 | 51 | 4 | 56 | 19 | 58 | 6 |
| Metrafenone | 58 | 6 | 60 | 13 | 60 | 8 | 58 | 3 | 35 | 6 | 41 | 3 | 59 | 8 | 69 | 8 | 47 | 5 | 49 | 3 | 65 | 18 | 60 | 8 |
| Napropamid | 53 | 19 | 70 | 12 | 58 | 9 | 59 | 1 | 67 | 9 | 63 | 7 | 65 | 16 | 84 | 8 | 54 | 6 | 50 | 6 | 57 | 18 | 52 | 8 |
| Norflurazon | 53 | 5 | 50 | 6 | 49 | 8 | 50 | 4 | 31 | 15 | 30 | 10 | 55 | 9 | 69 | 6 | 36 | 5 | 40 | 7 | 49 | 13 | 37 | 6 |
| Oxadiargyl | 77 | 14 | 67 | 10 | 63 | 8 | 61 | 6 | 57 | 18 | 60 | 9 | 67 | 11 | 75 | 3 | 59 | 9 | 54 | 8 | 79 | 27 | 64 | 19 |
| Oxycarboxim | 27 | 3 | 24 | 4 | 24 | 8 | 29 | 3 | 22 | 5 | 28 | 5 | 31 | 6 | 33 | 2 | 21 | 7 | 26 | 6 | 31 | 7 | 27 | 2 |
| Paclobutrazol | 108 | 5 | 105 | 14 | 76 | 10 | 59 | 5 | 50 | 12 | 60 | 11 | 102 | 6 | 97 | 5 | 53 | 12 | 53 | 8 | 60 | 26 | 55 | 12 |
| Pencycuron | 54 | 4 | 85 | 10 | 65 | 12 | 62 | 6 | 47 | 7 | 44 | 8 | 74 | 5 | 80 | 9 | 58 | 5 | 55 | 7 | 70 | 19 | 79 | 7 |
| Penthiopyrad | 67 | 9 | 67 | 18 | 68 | 8 | 61 | 3 | 52 | 7 | 54 | 15 | 70 | 6 | 71 | 4 | 56 | 12 | 51 | 4 | 79 | 24 | 83 | 7 |
| Pethoxamid | 52 | 9 | 52 | 6 | 71 | 10 | 64 | 8 | 68 | 7 | 61 | 7 | 85 | 11 | 90 | 5 | 55 | 6 | 54 | 10 | 64 | 21 | 59 | 6 |
| Phenthoate | 58 | 12 | 62 | 17 | 62 | 9 | 65 | 5 | 46 | 7 | 46 | 5 | 72 | 9 | 74 | 5 | 53 | 6 | 55 | 4 | 70 | 18 | 73 | 9 |
| Phosalone | 56 | 16 | 61 | 8 | 58 | 13 | 56 | 4 | 38 | 11 | 46 | 5 | 54 | 7 | 55 | 3 | 50 | 5 | 48 | 6 | 59 | 17 | 56 | 5 |
| Phosmet | 40 | 6 | 48 | 5 | 45 | 42 | 55 | 8 | 34 | 23 | 32 | 6 | 51 | 14 | 54 | 6 | 37 | 9 | 40 | 13 | 58 | 28 | 65 | 9 |
| Phosphamidon | 44 | 8 | 58 | 9 | 42 | 7 | 40 | 3 | 42 | 3 | 37 | 5 | 70 | 4 | 69 | 8 | 38 | 4 | 34 | 7 | 58 | 12 | 44 | 4 |
| Phoxim | 56 | 10 | 68 | 7 | 60 | 6 | 56 | 4 | 59 | 2 | 52 | 7 | 75 | 3 | 73 | 4 | 54 | 3 | 54 | 5 | 60 | 20 | 62 | 6 |
| Picolinafen | 56 | 3 | 59 | 7 | 58 | 15 | 61 | 4 | 33 | 8 | 47 | 4 | 59 | 6 | 65 | 4 | 51 | 7 | 51 | 8 | 65 | 17 | 67 | 5 |
| Picoxystrobin | 54 | 2 | 58 | 13 | 58 | 7 | 57 | 4 | 41 | 3 | 41 | 2 | 79 | 3 | 75 | 3 | 54 | 5 | 53 | 3 | 69 | 24 | 73 | 11 |
| Pinoxaden | 73 | 5 | 78 | 9 | 65 | 7 | 52 | 4 | 53 | 4 | 49 | 7 | 88 | 5 | 86 | 8 | 53 | 3 | 47 | 3 | 64 | 21 | 57 | 3 |

| | | | | | | | | | | | | | | | | | | | | | | | | |
|---|---|---|---|---|---|---|---|---|---|---|---|---|---|---|---|---|---|---|---|---|---|---|---|---|
| Piperonyl Butoxyde | 62 | 12 | 77 | 8 | 65 | 10 | 66 | 2 | 63 | 3 | 55 | 4 | 85 | 5 | 82 | 3 | 66 | 8 | 57 | 6 | 83 | 17 | 71 | 9 |
| Pirimiphos-ethyl | 57 | 7 | 60 | 7 | 65 | 6 | 63 | 3 | 56 | 1 | 50 | 3 | 66 | 11 | 68 | 5 | 61 | 2 | 58 | 6 | 64 | 19 | 64 | 3 |
| Pirimiphos-methyl | 63 | 5 | 62 | 7 | 69 | 7 | 62 | 4 | 63 | 2 | 52 | 7 | 91 | 10 | 70 | 3 | 64 | 3 | 60 | 5 | 68 | 28 | 63 | 8 |
| Prochloraz | 63 | 8 | 68 | 12 | 60 | 7 | 55 | 4 | 42 | 9 | 48 | 9 | 65 | 9 | 67 | 1 | 51 | 5 | 45 | 7 | 57 | 21 | 59 | 6 |
| Profenofos | 62 | 9 | 58 | 4 | 61 | 9 | 56 | 4 | 46 | 12 | 46 | 5 | 72 | 8 | 75 | 4 | 51 | 4 | 53 | 6 | 57 | 20 | 51 | 4 |
| Prometon | 37 | 8 | 43 | 10 | 55 | 8 | 56 | 4 | 31 | 3 | 25 | 3 | 67 | 5 | 67 | 8 | 45 | 3 | 46 | 6 | 60 | 17 | 47 | 6 |
| Prometryn | 68 | 4 | 71 | 5 | 69 | 5 | 69 | 2 | 50 | 5 | 48 | 3 | 67 | 3 | 64 | 4 | 56 | 8 | 56 | 6 | 57 | 27 | 59 | 6 |
| Propaphos | 65 | 11 | 66 | 11 | 71 | 14 | 69 | 5 | 50 | 4 | 48 | 3 | 83 | 6 | 83 | 14 | 57 | 5 | 58 | 4 | 65 | 17 | 60 | 10 |
| Propquinazid | 51 | 7 | 50 | 9 | 44 | 19 | 44 | 3 | 33 | 12 | 36 | 2 | 32 | 7 | 32 | 2 | 36 | 6 | 39 | 5 | 52 | 18 | 48 | 4 |
| Propargite | 64 | 6 | 67 | 9 | 67 | 9 | 68 | 3 | 69 | 9 | 61 | 3 | 55 | 6 | 58 | 4 | 59 | 3 | 58 | 5 | 73 | 18 | 67 | 5 |
| Propazine | 52 | 7 | 55 | 6 | 83 | 4 | 73 | 3 | 53 | 5 | 53 | 6 | 53 | 6 | 53 | 6 | 63 | 4 | 61 | 5 | 52 | 20 | 47 | 5 |
| Propiconazole | 58 | 11 | 61 | 10 | 58 | 4 | 55 | 6 | 53 | 10 | 55 | 6 | 70 | 5 | 73 | 6 | 54 | 3 | 51 | 4 | 60 | 16 | 55 | 4 |
| Propyzamide | 82 | 13 | 75 | 11 | 72 | 9 | 68 | 2 | 49 | 12 | 53 | 4 | 70 | 10 | 69 | 7 | 59 | 3 | 56 | 6 | 75 | 30 | 56 | 8 |
| Prosulfocarb | 73 | 14 | 63 | 16 | 84 | 8 | 71 | 5 | 69 | 4 | 64 | 6 | 68 | 7 | 68 | 8 | 66 | 8 | 61 | 4 | 77 | 26 | 62 | 6 |
| Pyraclostrobin | 58 | 8 | 65 | 7 | 58 | 7 | 55 | 5 | 47 | 6 | 45 | 4 | 67 | 6 | 66 | 3 | 51 | 4 | 49 | 4 | 67 | 19 | 68 | 5 |
| Pyrazophos | 69 | 1 | 74 | 8 | 62 | 10 | 62 | 2 | 47 | 11 | 49 | 5 | 85 | 9 | 90 | 7 | 52 | 3 | 55 | 4 | 77 | 21 | 72 | 8 |
| Pyridaben | 63 | 7 | 65 | 10 | 54 | 16 | 54 | 2 | 31 | 12 | 34 | 4 | 40 | 4 | 39 | 4 | 46 | 4 | 48 | 5 | 66 | 24 | 58 | 6 |
| Pyridafenthion | 74 | 19 | 90 | 8 | 59 | 14 | 55 | 6 | 39 | 16 | 35 | 4 | 77 | 12 | 78 | 6 | 47 | 8 | 44 | 5 | 68 | 19 | 64 | 8 |
| Pyridate | 67 | 5 | 59 | 6 | 45 | 10 | 37 | 6 | 42 | 3 | 23 | 4 | 50 | 4 | 36 | 3 | 83 | 5 | 66 | 6 | 64 | 19 | 54 | 6 |
| Rimsulfuron | 48 | 7 | 54 | 11 | 18 | 6 | 20 | 2 | 13 | 17 | 14 | 4 | 72 | 4 | 77 | 5 | 27 | 11 | 26 | 8 | 76 | 13 | 55 | 6 |
| Saflufenacil | 102 | 14 | 96 | 18 | 68 | 9 | 72 | 8 | 41 | 13 | 43 | 10 | 141 | 14 | 154 | 10 | 75 | 11 | 78 | 5 | 141 | 16 | 149 | 25 |
| Spinosyn-A | 71 | 6 | 70 | 13 | 60 | 7 | 60 | 5 | 72 | 4 | 71 | 3 | 70 | 8 | 69 | 8 | 49 | 4 | 50 | 7 | 74 | 18 | 67 | 4 |
| Spirotetramat | 87 | 9 | 91 | 14 | 63 | 10 | 56 | 6 | 50 | 31 | 54 | 6 | 156 | 4 | 144 | 7 | 51 | 4 | 46 | 5 | 94 | 17 | 84 | 5 |
| Spiroxamine | 73 | 10 | 80 | 9 | 70 | 10 | 56 | 3 | 56 | 7 | 53 | 5 | 96 | 5 | 96 | 6 | 64 | 2 | 55 | 5 | 69 | 24 | 68 | 11 |
| Sulfotep | 61 | 16 | 62 | 20 | 59 | 9 | 59 | 3 | 46 | 16 | 53 | 6 | 83 | 11 | 83 | 5 | 48 | 10 | 50 | 8 | 64 | 15 | 56 | 5 |
| Tebufenozide | 57 | 14 | 65 | 11 | 65 | 11 | 62 | 4 | 39 | 8 | 41 | 4 | 67 | 2 | 65 | 7 | 56 | 8 | 49 | 7 | 73 | 25 | 70 | 2 |
| Tebufenpyrad | 61 | 10 | 63 | 8 | 73 | 15 | 71 | 5 | 47 | 13 | 55 | 6 | 68 | 11 | 73 | 4 | 66 | 7 | 62 | 7 | 62 | 23 | 64 | 3 |
| Tepraloxydim | 71 | 3 | 72 | 7 | 51 | 6 | 45 | 8 | 35 | 13 | 37 | 11 | 63 | 7 | 73 | 4 | 44 | 9 | 42 | 3 | 72 | 24 | 64 | 7 |
| Terbufos | 72 | 7 | 72 | 11 | 74 | 16 | 64 | 5 | 69 | 13 | 66 | 14 | 63 | 8 | 63 | 9 | 71 | 13 | 66 | 5 | 89 | 23 | 65 | 5 |
| Thiobencarb | 65 | 8 | 65 | 11 | 69 | 12 | 62 | 7 | 55 | 8 | 57 | 13 | 72 | 6 | 68 | 5 | 57 | 12 | 58 | 4 | 65 | 20 | 60 | 6 |

| | | | | | | | | | | | | | | | | | | | | | | | | |
|---|---|---|---|---|---|---|---|---|---|---|---|---|---|---|---|---|---|---|---|---|---|---|---|---|
| Tolfenpyrad | 64 | 4 | 72 | 5 | 73 | 14 | 74 | 3 | 41 | 12 | 52 | 5 | 64 | 8 | 69 | 3 | 63 | 5 | 67 | 5 | 69 | 24 | 70 | 4 |
| Tralkoxydim | 60 | 6 | 61 | 6 | 60 | 14 | 56 | 3 | 44 | 8 | 48 | 4 | 29 | 14 | 56 | 9 | 53 | 6 | 53 | 8 | 55 | 19 | 55 | 5 |
| Triadimefon | 124 | 9 | 109 | 8 | 74 | 7 | 76 | 4 | 59 | 10 | 63 | 6 | 81 | 12 | 82 | 8 | 66 | 4 | 65 | 7 | 68 | 22 | 57 | 4 |
| Triallate | 104 | 14 | 62 | 10 | 64 | 19 | 63 | 8 | 64 | 14 | 58 | 7 | 37 | 18 | 65 | 9 | 62 | 13 | 56 | 8 | 0 | / | 59 | 11 |
| Tridemorph | 56 | 12 | 66 | 17 | 64 | 10 | 52 | 8 | 52 | 3 | 54 | 5 | 94 | 9 | 84 | 11 | 70 | 7 | 54 | 3 | 73 | 13 | 61 | 5 |
| Triforine | 78 | 17 | 79 | 5 | 63 | 8 | 54 | 4 | 34 | 22 | 39 | 18 | 75 | 8 | 80 | 1 | 50 | 14 | 49 | 9 | 32 | 44 | 101 | 17 |
| Zoxamide | 55 | 4 | 52 | 19 | 63 | 12 | 61 | 3 | 58 | 6 | 39 | 2 | 71 | 7 | 74 | 13 | 52 | 9 | 52 | 6 | 72 | 24 | 67 | 8 |

Table S3: LODs, LOQs and ME of the different extraction/cleanup QuEChERS method in the rapeseed samples.

| Molecules | Without clean up | | | Freezing | | | C18/PSA | | | EMR-lipid | | | Z-Sep | | | Z-Sep+ | | |
|---|---|---|---|---|---|---|---|---|---|---|---|---|---|---|---|---|---|---|
| | LOD (µg/kg) | LOQ (µg/kg) | ME (%) | LOD (µg/kg) | LOQ (µg/kg) | ME (%) | LOD (µg/kg) | LOQ (µg/kg) | ME (%) | LOD (µg/kg) | LOQ (µg/kg) | ME (%) | LOD (µg/kg) | LOQ (µg/kg) | ME (%) | LOD (µg/kg) | LOQ (µg/kg) | ME (%) |
| Acephate | 1.06 | 3.18 | -59 | 1.01 | 3.02 | 2 | 0.92 | 2.77 | -46 | 0.84 | 2.52 | -14 | 1.13 | 3.40 | -71 | 0.82 | 2.46 | -50 |
| Acetochlor | 1.52 | 4.57 | -39 | 1.09 | 3.28 | -27 | 1.10 | 3.30 | -12 | 0.84 | 2.51 | -14 | 1.25 | 3.76 | -41 | 1.05 | 3.14 | -60 |
| Acibenzolar-S-methyl | 0.76 | 2.27 | -22 | 1.26 | 3.77 | 8 | 1.22 | 3.67 | -6 | 1.33 | 3.99 | -13 | 1.96 | 5.87 | -31 | / | / | -72 |
| Alachlor | 1.07 | 3.21 | -36 | 1.01 | 3.03 | -19 | 1.09 | 3.28 | 4 | 0.81 | 2.43 | -37 | 1.24 | 3.71 | -28 | 1.19 | 3.58 | -60 |
| Aldicarb sulfone | 0.73 | 2.18 | -41 | 1.50 | 4.49 | -17 | 0.74 | 2.22 | -10 | 0.65 | 1.94 | -37 | / | / | -42 | 0.64 | 1.93 | -40 |
| Ametoctradin | 0.82 | 2.45 | -21 | 0.87 | 2.61 | -9 | 1.19 | 3.58 | 34 | 0.86 | 2.59 | -22 | 1.03 | 3.08 | -20 | 1.17 | 3.50 | -60 |
| Ametryn | 1.40 | 4.20 | -44 | 1.64 | 4.92 | -10 | / | / | 7 | 1.04 | 3.11 | -37 | 1.94 | 5.82 | -30 | 1.35 | 4.05 | -65 |
| Anilofos | 1.05 | 3.14 | -12 | 1.31 | 3.93 | -13 | 1.81 | 5.43 | 37 | 1.00 | 2.99 | -6 | 1.62 | 4.87 | -2 | 0.92 | 2.77 | -48 |
| Azaconazole | 0.75 | 2.25 | -24 | 1.19 | 3.58 | -9 | 1.90 | 5.69 | 38 | 1.01 | 3.04 | -11 | 1.46 | 4.38 | -11 | 1.11 | 3.34 | -65 |
| Azinphos-methyl | 0.68 | 2.04 | -34 | 1.31 | 3.93 | 14 | / | / | 19 | 0.93 | 2.79 | -32 | 1.69 | 5.06 | -13 | 1.14 | 3.42 | -49 |
| Aziprotryne | 0.91 | 2.73 | -24 | 0.85 | 2.54 | 4 | 1.07 | 3.22 | 32 | 0.83 | 2.49 | -22 | 1.08 | 3.24 | -20 | 1.01 | 3.03 | -68 |
| Azoxystrobin | 0.90 | 2.69 | -22 | 1.06 | 3.18 | -10 | 1.41 | 4.24 | 8 | 0.89 | 2.67 | -24 | 1.31 | 3.94 | -21 | 1.13 | 3.39 | -58 |
| Barban | 0.73 | 2.19 | -24 | 0.92 | 2.75 | -6 | 0.90 | 2.71 | 16 | 1.27 | 3.82 | -4 | 0.90 | 2.69 | -5 | / | / | -63 |
| Benalaxyl | 1.04 | 3.13 | -15 | 0.93 | 2.78 | 4 | 1.18 | 3.54 | 37 | 0.74 | 2.21 | -15 | 1.07 | 3.20 | -1 | 0.96 | 2.89 | -48 |
| Benoxacor | 1.08 | 3.25 | -17 | 1.02 | 3.07 | -1 | 1.64 | 4.91 | 12 | 0.91 | 2.72 | -40 | 1.36 | 4.09 | -12 | 1.06 | 3.17 | -48 |
| Bifenox | 0.99 | 2.96 | 14 | 1.23 | 3.70 | 52 | / | / | 99 | 1.36 | 4.08 | 30 | 1.43 | 4.29 | 31 | 1.30 | 3.89 | -54 |
| Bixafen | 1.05 | 3.15 | -25 | 1.07 | 3.20 | 13 | 1.18 | 3.54 | 21 | 0.93 | 2.80 | -10 | 1.51 | 4.54 | -6 | 0.97 | 2.91 | -53 |
| Boscalid | 0.82 | 2.45 | -29 | 0.68 | 2.03 | 2 | 1.80 | 5.40 | 24 | 0.76 | 2.29 | -6 | 0.75 | 2.25 | -25 | 0.58 | 1.75 | -55 |
| Bupirimate | 0.99 | 2.96 | -25 | 1.06 | 3.17 | -15 | 1.33 | 3.99 | 14 | 0.91 | 2.73 | -37 | 1.27 | 3.81 | -12 | 1.07 | 3.22 | -51 |
| Buprofezin | 1.15 | 3.44 | -77 | 0.98 | 2.94 | 5 | 1.08 | 3.23 | 29 | 0.90 | 2.69 | -11 | 1.05 | 3.16 | -9 | 1.07 | 3.21 | -53 |
| Chlorfenvinphos | 1.01 | 3.03 | -37 | 1.16 | 3.49 | -14 | 1.65 | 4.95 | 19 | 1.01 | 3.02 | -21 | 1.49 | 4.48 | -23 | 0.93 | 2.79 | -59 |

| | | | | | | | | | | | | | | | | | | |
|---|---|---|---|---|---|---|---|---|---|---|---|---|---|---|---|---|---|---|
| Chlorobromuron | 1.09 | 3.28 | -31 | 1.25 | 3.74 | -30 | 1.38 | 4.14 | -10 | 1.21 | 3.62 | -43 | 1.41 | 4.22 | -36 | 1.14 | 3.43 | -73 |
| Chlorfluazuron | 0.92 | 2.76 | -52 | 1.00 | 3.00 | -48 | 1.40 | 4.19 | -35 | 1.50 | 4.50 | -34 | 1.15 | 3.45 | -53 | 0.82 | 2.45 | -76 |
| Chlorpyrifos | 1.13 | 3.40 | -15 | 1.12 | 3.37 | 1 | 1.23 | 3.68 | 46 | 0.87 | 2.62 | -8 | 1.25 | 3.76 | -9 | 1.13 | 3.39 | -58 |
| Chlorpyrifos-methyl | 1.05 | 3.14 | -30 | 1.18 | 3.53 | -23 | 1.22 | 3.67 | -10 | 0.88 | 2.65 | -21 | 1.12 | 3.36 | -60 | 1.43 | 4.29 | -69 |
| Cinosulfuron | 1.33 | 4.00 | -29 | / | / | 32 | / | / | 54 | 0.58 | 1.75 | 2 | 1.99 | 5.96 | 21 | 0.84 | 2.51 | -34 |
| Climbazole | 1.05 | 3.14 | -43 | 1.37 | 4.11 | 5 | / | / | 29 | 0.82 | 2.46 | -28 | 1.78 | 5.34 | -15 | 1.03 | 3.10 | -59 |
| Clodinafop-propargyl | 0.93 | 2.80 | -4 | 1.08 | 3.23 | 13 | 1.60 | 4.79 | 25 | 0.85 | 2.54 | -7 | 1.35 | 4.06 | -12 | 0.89 | 2.67 | -42 |
| Clofentezine | 1.09 | 3.26 | -26 | 0.93 | 2.78 | 5 | 1.24 | 3.73 | 28 | 1.47 | 4.40 | -21 | 1.15 | 3.46 | -22 | 1.35 | 4.06 | -57 |
| Cloquintocet-mexyl | 1.01 | 3.04 | 70 | 0.94 | 2.82 | 18 | 1.08 | 3.24 | 90 | 0.91 | 2.72 | 51 | 1.05 | 3.16 | 58 | 1.13 | 3.39 | 87 |
| Coumaphos | 1.10 | 3.29 | -36 | 1.12 | 3.37 | -16 | 1.79 | 5.37 | 22 | 1.00 | 3.01 | -20 | 1.32 | 3.95 | -15 | 0.89 | 2.66 | -56 |
| Coumatetralyl | 1.50 | 4.49 | -30 | 1.25 | 3.76 | -5 | 1.46 | 4.39 | 10 | 0.95 | 2.84 | -37 | 1.84 | 5.53 | -20 | 1.23 | 3.69 | -58 |
| Crufomate | 1.02 | 3.07 | -13 | 0.81 | 2.42 | 4 | 1.07 | 3.20 | 37 | 0.74 | 2.21 | -11 | 0.99 | 2.98 | -14 | 1.05 | 3.15 | -51 |
| Cyanazine | 1.36 | 4.09 | -38 | 0.87 | 2.60 | -5 | / | / | 3 | 0.84 | 2.51 | -40 | 1.00 | 2.99 | -28 | 1.09 | 3.26 | -57 |
| Cycloxydim | 1.10 | 3.30 | -60 | 1.18 | 3.53 | 5 | 1.22 | 3.65 | 60 | 0.99 | 2.98 | 1 | 1.39 | 4.17 | -13 | 1.29 | 3.86 | -48 |
| Cyflumetofen | 0.88 | 2.64 | -27 | 0.78 | 2.35 | -23 | 0.82 | 2.45 | 24 | 0.67 | 2.01 | -22 | 0.82 | 2.46 | -28 | 0.79 | 2.36 | -62 |
| Cymiazole | 1.79 | 5.36 | -62 | 1.65 | 4.95 | -31 | 1.59 | 4.77 | -33 | 1.34 | 4.03 | -59 | 2.04 | 6.11 | -50 | 1.48 | 4.43 | -67 |
| Cyproconazole | 0.78 | 2.34 | -32 | 1.13 | 3.40 | 5 | 1.04 | 3.12 | 20 | 0.80 | 2.40 | -17 | 1.16 | 3.49 | -20 | 1.06 | 3.18 | -57 |
| Cyprodinil | 1.06 | 3.18 | -18 | 0.98 | 2.94 | 2 | 1.57 | 4.71 | 31 | 0.57 | 1.72 | -20 | 1.17 | 3.52 | -18 | / | / | -59 |
| Demeton-S-methyl sulfone | 1.40 | 4.20 | -55 | 1.09 | 3.28 | -16 | 1.35 | 4.04 | -9 | 1.14 | 3.41 | -39 | 1.17 | 3.50 | -40 | 1.17 | 3.50 | -61 |
| Desmetryn | 1.49 | 4.46 | -29 | 1.61 | 4.84 | 0 | / | / | 17 | 1.09 | 3.26 | -20 | 2.06 | 6.19 | -25 | 1.38 | 4.14 | -55 |
| Diafenthiuron | 0.91 | 2.73 | 26 | 1.17 | 3.52 | 22 | 0.70 | 2.11 | 22 | 0.95 | 2.86 | 15 | 1.49 | 4.47 | 27 | 1.07 | 3.20 | -46 |
| Dialifos | 1.14 | 3.43 | -30 | 1.27 | 3.82 | -2 | 1.94 | 5.81 | 28 | 1.16 | 3.49 | -14 | 1.39 | 4.16 | -14 | 1.05 | 3.14 | -66 |
| Diazinon | 1.17 | 3.50 | -21 | 0.94 | 2.83 | -7 | 1.17 | 3.50 | 28 | 0.92 | 2.77 | -21 | 1.08 | 3.25 | -9 | 0.88 | 2.63 | -54 |
| Diclofop-methyl | 1.05 | 3.16 | -91 | 1.18 | 3.54 | -58 | 1.59 | 4.78 | -86 | 1.06 | 3.18 | -91 | 1.39 | 4.18 | -91 | 1.00 | 3.00 | -95 |
| Diethofencarb | 1.11 | 3.33 | -25 | 1.15 | 3.46 | -13 | 1.65 | 4.95 | -4 | 0.77 | 2.32 | -19 | 1.44 | 4.33 | -21 | 1.13 | 3.38 | -54 |
| Difenoconazole | 0.85 | 2.54 | -41 | 0.87 | 2.62 | 1 | 0.83 | 2.48 | 38 | 0.77 | 2.32 | 5 | 0.97 | 2.92 | -13 | 0.84 | 2.52 | -54 |
| Diflufenican | 0.95 | 2.86 | 50 | 0.95 | 2.86 | 25 | 1.57 | 4.70 | 62 | 1.14 | 3.43 | 10 | 1.06 | 3.17 | -37 | 0.93 | 2.80 | -48 |

| | | | | | | | | | | | | | | | | | | |
|---|---|---|---|---|---|---|---|---|---|---|---|---|---|---|---|---|---|---|
| Dimethachlor | 1.16 | 3.47 | -33 | 1.09 | 3.28 | -18 | 1.58 | 4.75 | 10 | 0.98 | 2.93 | -29 | 1.39 | 4.18 | -18 | 0.94 | 2.81 | -56 |
| Dimethenamid | 1.15 | 3.46 | -33 | 1.12 | 3.37 | -14 | 1.34 | 4.02 | 0 | 0.95 | 2.84 | -29 | 1.45 | 4.34 | -26 | 1.12 | 3.37 | -58 |
| Dimethomorph | 0.80 | 2.40 | 53 | 1.08 | 3.24 | 13 | 2.16 | 6.49 | 41 | 0.83 | 2.48 | 11 | 1.34 | 4.03 | 2 | 0.91 | 2.74 | -47 |
| Dimoxystrobin | 1.00 | 3.01 | -16 | 0.98 | 2.95 | -10 | 1.44 | 4.33 | 30 | 0.88 | 2.65 | -13 | 1.27 | 3.81 | -13 | 0.93 | 2.79 | -55 |
| Dioxathion | 0.93 | 2.78 | -23 | 0.95 | 2.86 | -7 | 1.24 | 3.73 | 24 | 0.97 | 2.90 | -22 | 1.11 | 3.34 | -14 | 0.97 | 2.91 | -56 |
| Dipropetryn | 1.49 | 4.46 | -28 | 1.16 | 3.48 | 2 | 1.22 | 3.65 | 29 | 0.78 | 2.33 | -24 | 1.30 | 3.89 | -11 | 1.17 | 3.51 | -50 |
| Ditalimphos | 1.37 | 4.12 | -32 | 1.23 | 3.70 | -11 | 1.42 | 4.25 | 21 | 0.93 | 2.79 | -32 | 1.34 | 4.03 | -13 | 1.15 | 3.45 | -49 |
| Emamectin Ba | 0.79 | 2.36 | -51 | 0.86 | 2.59 | 21 | 0.65 | 1.96 | 71 | 0.72 | 2.17 | -9 | 0.98 | 2.93 | 12 | 0.66 | 1.99 | -47 |
| Emamectin Bb | 0.93 | 2.78 | -8 | 0.71 | 2.14 | 19 | 0.66 | 1.99 | 58 | 0.75 | 2.25 | -6 | 0.89 | 2.66 | 8 | 0.66 | 1.97 | -52 |
| Epoxiconazole | 1.12 | 3.37 | -9 | 0.93 | 2.78 | 34 | 1.17 | 3.51 | 48 | 0.64 | 1.92 | -17 | 1.08 | 3.23 | 18 | 1.01 | 3.04 | -51 |
| Etaconazole | 0.86 | 2.57 | -13 | 1.21 | 3.64 | 6 | 1.44 | 4.33 | 43 | 0.57 | 1.71 | 2 | 1.03 | 3.09 | -17 | / | / | -70 |
| Ethion | 1.19 | 3.56 | -25 | 1.05 | 3.14 | 2 | 1.10 | 3.30 | 24 | 1.10 | 3.30 | -24 | 1.23 | 3.69 | -17 | 0.93 | 2.80 | -57 |
| Ethofumesate | 1.05 | 3.15 | -42 | / | / | -24 | 1.25 | 3.75 | -6 | 0.93 | 2.79 | -33 | / | / | -32 | 0.89 | 2.66 | -58 |
| Ethoprophos | 1.37 | 4.12 | -22 | 1.06 | 3.17 | 18 | 1.18 | 3.53 | 36 | 0.80 | 2.41 | -31 | 1.29 | 3.88 | -7 | 1.11 | 3.33 | -46 |
| Etoxazole | 1.09 | 3.26 | 133 | 1.13 | 3.40 | 75 | 1.24 | 3.73 | 181 | 1.66 | 4.98 | 117 | 1.33 | 3.99 | 147 | 0.98 | 2.93 | 37 |
| Etrimfos | 1.07 | 3.20 | -22 | 1.00 | 2.99 | -1 | 1.07 | 3.20 | 24 | 0.90 | 2.70 | -11 | 1.10 | 3.29 | -8 | 0.98 | 2.95 | -58 |
| Famoxadone | 0.95 | 2.86 | -42 | 0.91 | 2.73 | -25 | 1.41 | 4.23 | 17 | 0.77 | 2.30 | -24 | 1.04 | 3.11 | -28 | 0.86 | 2.57 | -61 |
| Fenamiphos | 1.24 | 3.72 | -11 | 0.88 | 2.63 | -3 | 1.22 | 3.67 | 28 | 0.83 | 2.49 | -28 | 1.12 | 3.36 | -12 | 1.09 | 3.26 | -49 |
| Fenarimol | 0.97 | 2.91 | 16 | 0.90 | 2.71 | 26 | 0.90 | 2.70 | 86 | 0.70 | 2.11 | -5 | 1.25 | 3.75 | 36 | 1.37 | 4.10 | -27 |
| Fenazaquin | 1.08 | 3.25 | -20 | 1.16 | 3.47 | -6 | 1.40 | 4.19 | 24 | 1.14 | 3.42 | -21 | 1.26 | 3.77 | -18 | 1.18 | 3.55 | -58 |
| Fenbuconazole | 0.88 | 2.63 | 31 | 0.94 | 2.83 | 15 | 1.42 | 4.27 | 60 | 0.84 | 2.52 | -20 | 1.18 | 3.53 | 5 | 0.95 | 2.86 | -37 |
| Fenpropidin | 0.92 | 2.76 | -4 | 1.11 | 3.32 | 12 | 1.34 | 4.03 | 52 | 0.75 | 2.24 | -4 | 1.12 | 3.35 | 0 | 0.91 | 2.74 | -47 |
| Fenpropimorph | 1.09 | 3.26 | -22 | 1.12 | 3.36 | -12 | 1.95 | 5.85 | 26 | 0.77 | 2.30 | -17 | 1.32 | 3.96 | -21 | 1.35 | 4.04 | -46 |
| Fenpyroximate | 0.92 | 2.77 | 19 | 1.05 | 3.15 | 26 | 1.26 | 3.77 | 55 | 1.29 | 3.86 | 16 | 1.22 | 3.66 | 17 | 0.89 | 2.66 | -39 |
| Fensulfothion | 1.26 | 3.78 | -41 | 1.35 | 4.04 | -72 | / | / | -50 | 1.09 | 3.27 | -28 | 1.65 | 4.96 | -22 | 1.12 | 3.36 | -54 |
| Fensulfothion-oxon | 2.01 | 6.03 | -28 | 1.94 | 5.81 | -12 | / | / | 27 | 0.86 | 2.59 | -18 | / | / | -9 | 1.72 | 5.16 | -58 |
| Fensulfothion-sulfone | 0.74 | 2.21 | -30 | 1.33 | 3.99 | 7 | / | / | 22 | 0.88 | 2.63 | -18 | 1.67 | 5.00 | -17 | 1.02 | 3.05 | -46 |
| Fenthion | 1.13 | 3.40 | -10 | 0.99 | 2.97 | -2 | 1.08 | 3.23 | 37 | 0.95 | 2.86 | 5 | 1.10 | 3.29 | -14 | 1.10 | 3.30 | -56 |
| Fenthion sulfone | 1.51 | 4.52 | -46 | 2.19 | 6.57 | -34 | / | / | -14 | 1.08 | 3.23 | -42 | / | / | -45 | 1.03 | 3.10 | -60 |

| | | | | | | | | | | | | | | | | | | |
|---|---|---|---|---|---|---|---|---|---|---|---|---|---|---|---|---|---|---|
| Fenthion sulfoxide | 1.55 | 4.66 | -46 | 1.75 | 5.24 | -30 | / | / | -10 | 1.36 | 4.07 | -41 | 1.89 | 5.66 | -37 | 1.30 | 3.89 | -59 |
| Flamprop-isopropyl | 1.13 | 3.39 | -5 | 1.07 | 3.20 | 8 | 1.42 | 4.25 | 48 | 0.92 | 2.75 | -8 | 1.37 | 4.12 | 5 | 0.90 | 2.70 | -55 |
| Flazasulfuron | 0.68 | 2.03 | 16 | 2.07 | 6.21 | 10 | / | / | 60 | 0.85 | 2.55 | 30 | 1.64 | 4.91 | 19 | 0.67 | 2.00 | -45 |
| Florasulam | 1.17 | 3.51 | 16 | 1.17 | 3.52 | 87 | / | / | 90 | 0.75 | 2.26 | 6 | 0.90 | 2.69 | 23 | 0.73 | 2.18 | -17 |
| Flubendiamide | 0.97 | 2.92 | -18 | 1.09 | 3.27 | -9 | 0.85 | 2.55 | 23 | 0.84 | 2.53 | -12 | 1.17 | 3.50 | -14 | 0.86 | 2.59 | -60 |
| Flucycloxuron | 0.84 | 2.51 | -15 | 0.84 | 2.53 | 10 | 1.43 | 4.29 | 42 | 1.04 | 3.11 | -12 | 1.02 | 3.06 | -12 | 0.81 | 2.43 | -57 |
| Flufenacet | 1.00 | 3.01 | 43 | 0.74 | 2.22 | 32 | 1.36 | 4.07 | 81 | 0.93 | 2.78 | -1 | 0.93 | 2.79 | 17 | 0.64 | 1.91 | -50 |
| Flufenoxuron | 0.94 | 2.81 | -31 | 0.95 | 2.86 | -2 | 1.13 | 3.38 | 20 | 1.23 | 3.69 | -22 | 1.23 | 3.70 | -23 | 0.86 | 2.58 | -58 |
| Fluopicolide | 1.05 | 3.14 | -45 | 1.29 | 3.88 | 14 | 2.08 | 6.24 | 35 | 1.30 | 3.91 | -6 | 1.63 | 4.88 | -9 | 1.04 | 3.13 | -50 |
| Flurochloridon | 0.88 | 2.63 | -30 | 1.22 | 3.66 | -7 | 1.84 | 5.51 | 3 | 1.01 | 3.04 | -58 | 1.53 | 4.60 | -23 | 1.16 | 3.49 | -57 |
| Flurprimidol | 0.81 | 2.43 | -41 | 0.94 | 2.83 | -8 | 1.13 | 3.40 | 14 | 0.78 | 2.34 | -17 | 1.11 | 3.34 | -19 | 0.95 | 2.86 | -61 |
| Flurtamone | 1.03 | 3.08 | -21 | 1.21 | 3.63 | -10 | 1.87 | 5.61 | 19 | 0.80 | 2.39 | -22 | 1.54 | 4.63 | -18 | 1.07 | 3.22 | -54 |
| Flusilazole | 1.05 | 3.15 | 18 | 0.83 | 2.49 | 8 | 1.00 | 3.01 | 72 | 0.86 | 2.58 | 7 | 1.04 | 3.13 | 0 | 1.10 | 3.29 | -44 |
| Fluxapyroxad | 0.95 | 2.85 | -30 | 1.17 | 3.50 | -10 | / | / | 10 | 1.19 | 3.56 | -21 | 1.75 | 5.26 | -24 | 0.98 | 2.93 | -57 |
| Fluxastrobin | 1.66 | 4.98 | -20 | 1.28 | 3.85 | -10 | 1.31 | 3.93 | 6 | 0.91 | 2.72 | -45 | 1.92 | 5.75 | -27 | 1.17 | 3.52 | -57 |
| Fonofos | 0.98 | 2.95 | -23 | 0.76 | 2.28 | -1 | 0.90 | 2.69 | 83 | 0.82 | 2.47 | 29 | 0.81 | 2.44 | -5 | 0.75 | 2.26 | -56 |
| Foramsulfuron | 0.71 | 2.14 | -89 | / | / | -91 | / | / | -85 | / | / | -37 | 1.58 | 4.75 | -89 | 0.78 | 2.35 | -93 |
| Forchlofenuron | 0.99 | 2.97 | -32 | 0.95 | 2.84 | -5 | / | / | 14 | 1.05 | 3.15 | -18 | 1.11 | 3.34 | -29 | 0.71 | 2.14 | -61 |
| Furmecyclox | 1.34 | 4.03 | -24 | 0.97 | 2.92 | 0 | 1.27 | 3.81 | 32 | 1.03 | 3.08 | -18 | 1.14 | 3.41 | -8 | 1.21 | 3.64 | -50 |
| Haloxyfop-methyl | 0.95 | 2.86 | -55 | 0.96 | 2.88 | 5 | 1.56 | 4.68 | 55 | 0.88 | 2.64 | -10 | 1.15 | 3.45 | 2 | 0.89 | 2.66 | -63 |
| Heptenophos | 0.68 | 2.04 | -45 | 1.11 | 3.32 | 9 | 1.42 | 4.27 | 17 | 0.87 | 2.61 | -36 | 1.42 | 4.26 | -16 | 0.94 | 2.82 | -50 |
| Hexazinone | 1.78 | 5.34 | -45 | 1.98 | 5.95 | -26 | / | / | -8 | 1.44 | 4.33 | -41 | / | / | -34 | 1.59 | 4.77 | -59 |
| Hexythiazox | 1.27 | 3.82 | -25 | 1.17 | 3.52 | -1 | 1.80 | 5.39 | 35 | 1.47 | 4.41 | -16 | 1.35 | 4.06 | -14 | 1.19 | 3.56 | -57 |
| Imazalil | 1.42 | 4.25 | -37 | 1.21 | 3.64 | -6 | 1.51 | 4.53 | 8 | 0.81 | 2.44 | -44 | 1.49 | 4.47 | -26 | 0.96 | 2.89 | -62 |
| Imazosulfuron | 0.71 | 2.14 | -3 | 1.21 | 3.64 | -15 | 1.81 | 5.44 | 21 | 0.63 | 1.88 | -9 | 0.81 | 2.44 | -9 | 0.87 | 2.61 | -70 |
| Imibenconazole | 1.09 | 3.26 | -6 | 0.60 | 1.79 | 14 | 1.15 | 3.45 | 64 | 1.19 | 3.58 | -8 | 0.78 | 2.33 | -3 | 1.03 | 3.08 | -51 |
| Indoxacarb | 0.68 | 2.05 | 15 | 0.73 | 2.20 | 24 | 1.45 | 4.34 | 50 | 0.63 | 1.89 | -59 | 0.76 | 2.27 | 27 | 0.57 | 1.71 | -48 |
| Ipconazole | 0.83 | 2.50 | 6 | 0.94 | 2.81 | 20 | 1.27 | 3.82 | 57 | 0.58 | 1.73 | -5 | 0.64 | 1.91 | -3 | 0.92 | 2.76 | -41 |
| Isofenphos | 1.10 | 3.29 | -21 | 0.73 | 2.19 | -2 | 1.67 | 5.00 | -59 | 0.87 | 2.61 | -11 | 0.80 | 2.40 | -11 | 1.04 | 3.12 | -52 |

| | | | | | | | | | | | | | | | | | | |
|---|---|---|---|---|---|---|---|---|---|---|---|---|---|---|---|---|---|---|
| Isofenphos-methyl | 0.93 | 2.78 | -20 | 1.14 | 3.42 | -8 | 1.90 | 5.71 | 40 | 0.92 | 2.77 | -19 | 1.27 | 3.80 | -9 | 0.91 | 2.74 | -52 |
| Isopyrazam | 1.00 | 3.00 | -17 | 0.95 | 2.85 | 6 | 1.23 | 3.70 | 44 | 0.85 | 2.55 | -7 | 1.08 | 3.24 | -15 | 0.83 | 2.49 | -57 |
| Isoxaflutole | 0.70 | 2.09 | 19 | 0.91 | 2.73 | -42 | 1.06 | 3.19 | 6 | / | / | 0 | 0.84 | 2.53 | 2 | 0.59 | 1.78 | -38 |
| Kresoxim-methyl | 0.95 | 2.84 | -14 | 0.92 | 2.76 | 0 | 1.14 | 3.43 | 46 | 0.79 | 2.38 | -14 | 1.26 | 3.77 | -13 | 1.02 | 3.05 | -53 |
| Linuron | 1.30 | 3.91 | -34 | 1.09 | 3.27 | -6 | 1.44 | 4.31 | 23 | 1.01 | 3.03 | -20 | 1.46 | 4.39 | -7 | 0.88 | 2.65 | -65 |
| Malaoxon | 1.78 | 5.35 | -47 | 1.35 | 4.05 | -38 | 1.85 | 5.54 | -28 | 1.04 | 3.12 | -45 | 1.48 | 4.45 | -49 | / | / | -70 |
| Malathion | 1.04 | 3.12 | -23 | 0.98 | 2.95 | 12 | 1.53 | 4.60 | 2 | 0.89 | 2.66 | -36 | 1.16 | 3.47 | -34 | 0.98 | 2.93 | -62 |
| Mecarbam | 1.04 | 3.13 | -20 | 1.38 | 4.13 | 8 | 1.75 | 5.26 | 33 | 0.90 | 2.69 | -37 | 1.73 | 5.18 | 0 | 1.04 | 3.12 | -54 |
| Mepanipyrim | 1.34 | 4.03 | -27 | 0.93 | 2.78 | -4 | 1.27 | 3.82 | 39 | 1.01 | 3.02 | -26 | 1.17 | 3.51 | -24 | 1.42 | 4.26 | -47 |
| Metaflumizone | 0.78 | 2.33 | -6 | 0.84 | 2.52 | 18 | 1.03 | 3.10 | 72 | 0.75 | 2.26 | -8 | 0.97 | 2.90 | -7 | 0.59 | 1.76 | -51 |
| Metalaxyl | 1.03 | 3.10 | -32 | 1.05 | 3.16 | 1 | 1.65 | 4.94 | 25 | 0.70 | 2.10 | -33 | 1.27 | 3.81 | -17 | 0.83 | 2.50 | -56 |
| Metazachlor | 1.75 | 5.25 | -40 | 1.35 | 4.05 | -10 | 1.95 | 5.86 | 11 | 1.00 | 3.00 | -30 | 1.59 | 4.78 | -29 | 1.17 | 3.50 | -54 |
| Methabenzthiazuron | 1.38 | 4.13 | -32 | 1.12 | 3.36 | -5 | 1.58 | 4.73 | -49 | 1.22 | 3.65 | -30 | 1.48 | 4.43 | -20 | 1.13 | 3.38 | -59 |
| Methidathion | 1.76 | 5.27 | -49 | 1.34 | 4.02 | -14 | 1.48 | 4.44 | 0 | 1.49 | 4.48 | -40 | 1.68 | 5.04 | -20 | 1.36 | 4.09 | -54 |
| Methiocarb | 1.18 | 3.55 | -27 | 1.04 | 3.12 | -18 | 1.13 | 3.39 | 0 | 1.02 | 3.06 | -28 | 1.35 | 4.05 | -30 | 0.77 | 2.30 | -68 |
| Methoprotryne | 1.31 | 3.93 | -25 | 1.14 | 3.43 | -8 | 1.55 | 4.65 | 28 | 0.82 | 2.46 | -26 | 1.37 | 4.10 | -21 | 1.11 | 3.32 | -58 |
| Methoxyfenozide | 1.26 | 3.77 | -20 | 1.34 | 4.01 | 20 | 1.81 | 5.43 | 44 | 1.24 | 3.73 | -3 | 1.73 | 5.20 | -13 | 1.09 | 3.28 | -47 |
| Metobromuron | 1.21 | 3.64 | -44 | 1.55 | 4.64 | -16 | 2.16 | 6.49 | -7 | 1.03 | 3.09 | -41 | 1.82 | 5.46 | -44 | 1.11 | 3.32 | -63 |
| Metolachlor | 1.27 | 3.82 | -24 | 1.08 | 3.24 | -19 | 1.44 | 4.33 | 16 | 0.85 | 2.56 | -23 | 1.32 | 3.97 | -19 | 1.18 | 3.55 | -57 |
| Metrafenone | 1.16 | 3.47 | -16 | 1.12 | 3.36 | 9 | 1.90 | 5.69 | 45 | 1.14 | 3.41 | -8 | 1.42 | 4.26 | -1 | 1.03 | 3.09 | -54 |
| Napropamid | 1.26 | 3.79 | 22 | 1.14 | 3.43 | -1 | 1.00 | 2.99 | 37 | 1.02 | 3.06 | -33 | 1.24 | 3.73 | -7 | 1.17 | 3.51 | -51 |
| Norflurazon | 1.25 | 3.75 | -66 | 1.37 | 4.11 | -52 | 2.15 | 6.46 | -35 | 1.20 | 3.61 | -42 | 1.85 | 5.56 | -57 | 1.36 | 4.09 | -77 |
| Oxadiargyl | 0.87 | 2.60 | -17 | 1.06 | 3.18 | -12 | 1.16 | 3.49 | 11 | 0.99 | 2.97 | -11 | 1.12 | 3.37 | -2 | 0.84 | 2.53 | -57 |
| Oxycarboxim | / | / | -80 | / | / | -71 | / | / | -72 | 2.13 | 6.39 | -59 | / | / | -71 | 2.13 | 6.40 | -82 |
| Paclobutrazol | 0.72 | 2.17 | 10 | 0.88 | 2.64 | 17 | 1.32 | 3.96 | 59 | 0.65 | 1.96 | -1 | 1.25 | 3.75 | 9 | 1.11 | 3.32 | -62 |
| Pencycuron | 1.24 | 3.73 | -29 | 1.03 | 3.10 | -1 | 1.42 | 4.25 | 29 | 0.90 | 2.69 | -12 | 1.15 | 3.44 | -17 | 0.95 | 2.86 | -54 |
| Penthiopyrad | 1.00 | 3.00 | -13 | 0.98 | 2.95 | -1 | 1.28 | 3.84 | 30 | 0.95 | 2.86 | -6 | 1.20 | 3.59 | -10 | 0.84 | 2.52 | -48 |
| Pethoxamid | 1.27 | 3.82 | -16 | 0.93 | 2.80 | -5 | 0.98 | 2.95 | 18 | 0.78 | 2.34 | -43 | 1.21 | 3.64 | -17 | 1.04 | 3.11 | -58 |
| Phenthoate | 1.14 | 3.43 | -19 | 1.07 | 3.21 | 14 | 1.44 | 4.32 | 39 | 0.92 | 2.77 | -19 | 1.26 | 3.77 | -6 | 0.95 | 2.84 | -60 |

| | | | | | | | | | | | | | | | | | | |
|---|---|---|---|---|---|---|---|---|---|---|---|---|---|---|---|---|---|---|
| Phosalone | 1.20 | 3.59 | -32 | 1.15 | 3.45 | -4 | 1.74 | 5.23 | 36 | 1.24 | 3.71 | -17 | 1.32 | 3.97 | -11 | 1.13 | 3.39 | -64 |
| Phosmet | 1.66 | 4.99 | -93 | 1.47 | 4.41 | -68 | 1.94 | 5.83 | -84 | 1.29 | 3.88 | -49 | 1.79 | 5.36 | -88 | 1.16 | 3.47 | -94 |
| Phosphamidon | 1.52 | 4.57 | -52 | 1.57 | 4.72 | -35 | 1.60 | 4.79 | -13 | 0.95 | 2.86 | -44 | 1.74 | 5.22 | -46 | 1.14 | 3.43 | -70 |
| Phoxim | 1.19 | 3.57 | -31 | 1.11 | 3.32 | 1 | 1.14 | 3.41 | 24 | 0.89 | 2.67 | -13 | 1.24 | 3.73 | -13 | 1.10 | 3.31 | -62 |
| Picolinafen | 1.20 | 3.59 | -14 | 1.15 | 3.44 | 12 | 2.04 | 6.12 | 30 | 1.12 | 3.37 | -16 | 1.32 | 3.95 | -5 | 1.03 | 3.10 | -51 |
| Picoxystrobin | 1.23 | 3.70 | -20 | 1.16 | 3.47 | 0 | 1.62 | 4.86 | 29 | 0.84 | 2.53 | -12 | 1.24 | 3.73 | -22 | 0.96 | 2.89 | -41 |
| Pinoxaden | 0.92 | 2.75 | -11 | 1.03 | 3.10 | -5 | 1.26 | 3.77 | 32 | 0.76 | 2.28 | -8 | 1.26 | 3.77 | -48 | 1.03 | 3.10 | -48 |
| Piperonyl Butoxyde | 1.07 | 3.21 | -10 | 1.02 | 3.07 | 2 | 1.06 | 3.18 | 28 | 0.78 | 2.35 | -8 | 1.01 | 3.02 | -8 | 0.80 | 2.41 | -16 |
| Pirimiphos-ethyl | 1.18 | 3.53 | -13 | 1.02 | 3.06 | 9 | 1.19 | 3.57 | 32 | 1.02 | 3.05 | -2 | 1.09 | 3.28 | -4 | 1.04 | 3.13 | -39 |
| Pirimiphos-methyl | 1.06 | 3.18 | -31 | 0.96 | 2.88 | -7 | 1.05 | 3.16 | 21 | 0.73 | 2.20 | -18 | 1.04 | 3.13 | -21 | 0.99 | 2.96 | -56 |
| Prochloraz | 1.05 | 3.16 | -23 | 1.11 | 3.34 | -7 | 1.58 | 4.74 | 41 | 1.03 | 3.10 | -20 | 1.31 | 3.93 | 10 | 1.16 | 3.49 | -56 |
| Profenofos | 1.08 | 3.24 | 10 | 1.10 | 3.29 | 32 | 1.44 | 4.33 | 53 | 0.93 | 2.79 | 1 | 1.31 | 3.94 | 3 | 1.17 | 3.51 | -48 |
| Prometon | 1.79 | 5.37 | -37 | 1.22 | 3.67 | -9 | 2.15 | 6.46 | 11 | 1.00 | 2.99 | -29 | 1.47 | 4.40 | -26 | 1.11 | 3.32 | -63 |
| Prometryn | 0.99 | 2.96 | -14 | 0.97 | 2.90 | -2 | 1.34 | 4.03 | 20 | 0.99 | 2.98 | -21 | 1.19 | 3.56 | -23 | 1.17 | 3.51 | -53 |
| Propaphos | 1.02 | 3.07 | -10 | 0.94 | 2.81 | 3 | 1.34 | 4.03 | 47 | 0.81 | 2.42 | 1 | 1.17 | 3.51 | 8 | 1.02 | 3.06 | -39 |
| Propquinazid | 1.31 | 3.92 | -4 | 1.53 | 4.59 | 7 | 2.02 | 6.07 | 30 | 2.09 | 6.27 | -1 | 1.84 | 5.52 | 0 | 1.29 | 3.86 | -46 |
| Propargite | 1.04 | 3.11 | -23 | 0.99 | 2.98 | 2 | 0.97 | 2.90 | 24 | 1.21 | 3.63 | -18 | 1.14 | 3.41 | -15 | 0.92 | 2.75 | -54 |
| Propazine | 1.28 | 3.85 | -28 | 0.80 | 2.41 | -9 | 1.27 | 3.80 | 11 | 1.26 | 3.77 | -31 | 1.06 | 3.17 | -21 | 1.29 | 3.86 | -55 |
| Propiconazole | 1.15 | 3.46 | -26 | 1.15 | 3.45 | -12 | 1.26 | 3.79 | 21 | 0.95 | 2.86 | -94 | 1.23 | 3.68 | -16 | 1.12 | 3.35 | -53 |
| Propyzamide | 0.81 | 2.42 | -20 | 0.92 | 2.76 | 13 | 1.35 | 4.06 | 4 | 0.95 | 2.86 | -29 | 1.14 | 3.41 | -33 | 0.89 | 2.67 | -63 |
| Prosulfocarb | 0.91 | 2.72 | 161 | 0.79 | 2.37 | 51 | 0.96 | 2.88 | 30 | 0.98 | 2.95 | 71 | 1.01 | 3.03 | 65 | 0.86 | 2.59 | 18 |
| Pyraclostrobin | 1.16 | 3.47 | -29 | 1.16 | 3.48 | -2 | 1.42 | 4.25 | -87 | 1.00 | 3.01 | -24 | 1.31 | 3.94 | -2 | 1.00 | 3.00 | -49 |
| Pyrazophos | 0.97 | 2.92 | -15 | 1.08 | 3.23 | -1 | 1.43 | 4.28 | 45 | 0.78 | 2.35 | -4 | 1.28 | 3.83 | 1 | 0.87 | 2.61 | -10 |
| Pyridaben | 1.06 | 3.19 | -29 | 1.23 | 3.69 | 3 | 2.16 | 6.47 | 34 | 1.68 | 5.04 | -12 | 1.45 | 4.35 | -9 | 1.01 | 3.04 | -55 |
| Pyridafenthion | 0.90 | 2.69 | -15 | 1.13 | 3.39 | 0 | 1.73 | 5.18 | 28 | 0.86 | 2.58 | 2 | 1.43 | 4.30 | 2 | 0.99 | 2.96 | -42 |
| Pyridate | 1.00 | 2.99 | -22 | 1.47 | 4.41 | -68 | 1.58 | 4.74 | -27 | 1.34 | 4.03 | -21 | 0.81 | 2.42 | -19 | 1.04 | 3.13 | -58 |
| Rimsulfuron | 1.40 | 4.21 | -15 | / | / | -16 | / | / | 15 | 0.93 | 2.78 | -5 | / | / | -3 | 0.87 | 2.62 | -47 |
| Saflufenacil | 0.68 | 2.05 | 10 | 0.98 | 2.94 | -8 | 1.64 | 4.93 | 54 | / | / | -19 | 0.89 | 2.67 | 14 | 0.94 | 2.81 | -63 |
| Spinosyn-A | 0.94 | 2.82 | -20 | 1.12 | 3.36 | -1 | 0.93 | 2.79 | 36 | 0.95 | 2.86 | -18 | 1.36 | 4.08 | -26 | 0.90 | 2.70 | -62 |
| Spirotetramat | 0.77 | 2.30 | 0 | 1.06 | 3.17 | -5 | 1.34 | 4.03 | 35 | / | / | -16 | 1.32 | 3.95 | -1 | 0.71 | 2.12 | -45 |

| | | | | | | | | | | | | | | | | | | |
|---|---|---|---|---|---|---|---|---|---|---|---|---|---|---|---|---|---|---|
| Spiroxamine | 0.91 | 2.72 | -10 | 0.95 | 2.86 | 10 | 1.19 | 3.57 | 35 | 0.70 | 2.09 | -8 | 1.04 | 3.11 | -7 | 0.96 | 2.88 | -48 |
| Sulfotep | 1.10 | 3.31 | -19 | 1.12 | 3.36 | -7 | 1.44 | 4.32 | 35 | 0.80 | 2.40 | 1 | 1.38 | 4.14 | -14 | 1.05 | 3.14 | -51 |
| Tebufenozide | 1.17 | 3.52 | -13 | 1.03 | 3.09 | 4 | 1.71 | 5.13 | -98 | 1.00 | 3.00 | -11 | 1.19 | 3.58 | -6 | 0.92 | 2.76 | -51 |
| Tebufenpyrad | 1.10 | 3.29 | -23 | 0.91 | 2.73 | -5 | 1.41 | 4.23 | 20 | 0.97 | 2.92 | -16 | 1.02 | 3.05 | -19 | 1.08 | 3.25 | -62 |
| Tepraloxydim | 0.94 | 2.82 | -51 | 1.31 | 3.93 | -11 | 1.89 | 5.67 | 17 | 1.05 | 3.16 | -7 | 1.51 | 4.53 | 4 | 0.92 | 2.77 | -51 |
| Terbufos | 0.93 | 2.79 | -9 | 0.89 | 2.68 | 22 | 0.96 | 2.88 | 22 | 1.06 | 3.19 | 1 | 0.94 | 2.82 | 7 | 0.75 | 2.26 | -49 |
| Thiobencarb | 1.03 | 3.09 | -11 | 0.97 | 2.91 | 6 | 1.22 | 3.65 | 6 | 0.92 | 2.76 | 7 | 1.17 | 3.51 | -10 | 1.03 | 3.08 | -57 |
| Tolfenpyrad | 1.05 | 3.14 | -23 | 0.91 | 2.73 | 6 | 1.61 | 4.83 | 6 | 1.05 | 3.14 | -12 | 1.06 | 3.17 | -6 | 0.96 | 2.89 | -55 |
| Tralkoxydim | 1.11 | 3.34 | -18 | 1.11 | 3.32 | 1 | 1.50 | 4.50 | 1 | / | / | -10 | 1.26 | 3.79 | -5 | 1.20 | 3.61 | -56 |
| Triadimefon | 0.82 | 2.47 | -22 | 0.90 | 2.69 | -7 | 1.14 | 3.41 | -7 | 0.82 | 2.47 | -17 | 1.00 | 3.01 | -31 | 0.98 | 2.93 | -56 |
| Triallate | 0.69 | 2.07 | -71 | 1.04 | 3.11 | -89 | 1.04 | 3.11 | -39 | 1.78 | 5.34 | -70 | 1.07 | 3.21 | -91 | / | / | -96 |
| Tridemorph | 1.19 | 3.58 | -22 | 1.04 | 3.13 | 2 | 1.28 | 3.85 | 2 | 0.71 | 2.14 | -11 | 0.95 | 2.85 | -7 | 0.92 | 2.76 | -56 |
| Triforine | 0.86 | 2.57 | -30 | 1.06 | 3.18 | -30 | 1.97 | 5.90 | -30 | 0.88 | 2.65 | -34 | 1.33 | 4.00 | -23 | 2.10 | 6.30 | -68 |
| Zoxamide | 1.20 | 3.61 | -27 | 1.06 | 3.17 | -8 | 1.16 | 3.48 | -8 | 0.94 | 2.81 | -21 | 1.29 | 3.86 | -9 | 0.92 | 2.77 | -54 |
